# What are the essential components to implement individual-focused interventions for well-being and burnout in critical care healthcare professionals? A realist expert opinion

**DOI:** 10.3389/fpsyg.2022.991946

**Published:** 2022-09-28

**Authors:** Nurul B. B. Adnan, Claire Baldwin, Hila A. Dafny, Diane Chamberlain

**Affiliations:** Caring Futures Institute, College of Nursing and Health Sciences, Flinders University, Bedford Park, SA, Australia

**Keywords:** critical care, healthcare professionals, well-being (I31), burnout—professional, individual interventions

## Abstract

**Background:**

This study aimed to determine what, how, and under what circumstances individual-focused interventions improve well-being and decrease burnout for critical care healthcare professionals.

**Method:**

This realist approach, expert opinion interview, was guided by the Realist And Meta-narrative Evidence Synthesis: Evolving Standards II (RAMESES II) guidelines. Semi-structured interviews with critical care experts were conducted to ascertain current and nuanced information on a set of pre-defined individual interventions summarized from a previous umbrella review. The data were appraised, and relationships between context, mechanisms, and outcomes were extracted, which created theory prepositions that refined the initial program theory.

**Results:**

A total of 21 critical care experts were individually interviewed. By understanding the complex interplay between organizational and personal factors that influenced intervention uptake, it was possible to decipher the most likely implementable intervention for critical care healthcare professionals. The expert recommendation suggested that interventions should be evidence-based, accessible, inclusive, and collaborative, and promote knowledge and skill development. Unique mechanisms were also required to achieve the positive effects of the intervention due to the presence of contextual factors within critical care settings. Mechanisms identified in this study included the facilitation of self-awareness, self-regulation, autonomy, collaboration, acceptance, and inclusion (to enable a larger reach to different social groups).

**Conclusion:**

This validation of a theoretical understanding of intervention that addressed well-being and burnout in critical care healthcare professionals by expert opinion demonstrated essential mechanisms and contextual factors to consider when designing and implementing interventions. Future research would benefit by piloting individual interventions and integrating these new theoretical findings to understand better their effectiveness for future translation into the “real-world” setting.

## Introduction

Workload and work-life balance are central to well-being, alongside the experiences of workplace support, respect, and feeling valued (Jarden et al., 2019). Well-being can be conceptualized as a spectrum, where high well-being denotes happiness and flourishing (Hall et al., 2016). Conversely, low well-being encompasses components of increase anxiety and depression (Hall et al., 2016). The concept of well-being has two facets—hedonic and eudaimonic well-being (Keyes, 2002). Hedonic well-being encompasses emotional components such as life satisfaction, happiness, and balance between positive and negative affect (Keyes, 2002; Schlieper, 2021). Eudaimonic well-being involves psychological and social components such as personal growth, autonomy, positive relations, and social integration and acceptance (Schotanus-Dijkstra et al., 2016). Critical Care Healthcare Professionals (CCHP) displaying both hedonic and eudaimonic well-being are viewed as “flourishing” within the workplace (Keyes, 2002; Keyes and Haidt, 2010). Being in a state of flourishing is a favorable form of mental health and describes the subjective estimate of one’s perceptions and evaluations of their life (in terms of social, emotional, and psychological functioning; Schotanus-Dijkstra et al., 2016; Schlieper, 2021). Schotanus-Dijkstra et al. (2016) reported that individuals who flourished within the workplace tended to have excellent physical and mental health and could cope with challenges both within and outside of work compared to people who were not flourishing (Schotanus-Dijkstra et al., 2016). The concept of flourishing is composed of varying factors that may play a role in counteracting high-stress levels, emotional exhaustion, and subsequently burnout experiences (Berend et al., 2020).

Burnout is defined as a negative reaction to chronic occupational stressors, where there is a misfit between the individual’s needs, values, and job performance (Galletta et al., 2016). Maslach et al. (1996) identified burnout as a psychological syndrome characterized by emotional exhaustion, depersonalization, and personal inefficacy (Maslach et al., 1996). Emotional exhaustion is relative to the employee’s stress experiences and result in decreased physical and emotional resources (Maslach et al., 1997; Leiter and Maslach, 2005; Taris et al., 2017). Maslach et al. (1997) suggested that emotional exhaustion diminished self-initiative and engagement and progressively decreased the capacity for demanding work (Maslach et al., 1997; Leiter and Maslach, 2005; Taris et al., 2017). Similarly, cynicism is caused by the overload of exhaustion and concerns to reactions of work detachment (Maslach et al., 1996; Portoghese et al., 2014). It facilitates an environment for emotional involvement within the workplace and contributes to the loss of enthusiasm (Leiter and Maslach, 2005). The third component of professional inefficacy can be perceived as decreased productivity, ineffectiveness, and lack of achievement within the workplace (Leiter and Maslach, 2005). Perceived professional inefficacy can be categorized as a personality characteristic (Shirom, 2003), whereas both emotional exhaustion and cynicism can be recognized as two fundamental core components of burnout (Bakker et al., 2002; González-Romá et al., 2006).

Critical Care Healthcare Professionals (CCHP) are at high risk of distress and burnout due to their highly demanding and challenging work conditions (Galletta et al., 2016). These conditions enforce certain mental and social demands, negatively affecting their health and well-being (Galletta et al., 2016; Rattray et al., 2021). Example conditions included the constant care of high acuity patients, high workload and time pressures, reduced social support, and frequent unexpected critical events that often lead to suffering and death (Galletta et al., 2016; Rattray et al., 2021). For these reasons, critical care units are considered high-strain workplaces that predisposed workers to adaptation disorders and job dissatisfaction (Galletta et al., 2016; Rattray et al., 2021). Burnout experiences are common among critical care employees, having reached the epidemic level (Gomez et al., 2020). The COVID-19 pandemic and the healthcare system’s response have placed immense and unprecedented strain on the critical care workforce (Ripp et al., 2020). Being at the front line has meant that CCHP has been forced to meet sudden and dramatic rises in workload and demands, namely, expanding critical care provisions (Ripp et al., 2020). This experience has produced its own types of psychological stressors, including concerns regarding a lack of Personal Protective Equipment, contracting the virus and risking exposure to family and friends, as well as increased adverse patient outcomes and mortality (Gomez et al., 2020; Greenberg et al., 2021). These working conditions can adversely affect the mental health of CCHP, including moral injury and mental health diagnoses such as depression and post-traumatic stress disorder (Gomez et al., 2020; Greenberg et al., 2021).

Depression and post-traumatic stress disorder was identified as being more prevalent in critical care physicians and nurses with burnout syndrome (Kerlin et al., 2020). The effects of burnout can have wide-ranging effects on both the individual and the safety of patient care (Kerlin et al., 2020). An observational prospective multicenter study (31 intensive care units and 1,500 employees) determined that depression in CCHP was an independent risk factor for errors within the workplace (Garrouste-Orgeas et al., 2015). The study also reported a relationship between productivity and burnout—an increase in sick days and intent to leave the job as burnout experiences increased (Dewa et al., 2014; Garrouste-Orgeas et al., 2015).

In 2016, the Critical Care Societies Collaborative announced *a “call for action”* that encouraged stakeholder groups to promote well-being among CCHPs by protecting their mental and physical health (Moss et al., 2016). In addition, the Critical Care Societies Collaborative anticipated that novel methods for addressing burnout and well-being would be discovered with the assistance of stakeholders that may, in turn, shape regulations, promote quality patient care and decrease healthcare costs (associated with turnover; Moss et al., 2016). Stakeholders may be defined as a person or entity with a conceivable or declared stake or interest in a policy concern (Garthwaite et al., 2005). In this paper, we focused on expert stakeholders (herein referred to as experts) being people whose knowledge in the subject of interest (critical care workforce) has been earned through training or education and life experience (Garthwaite et al., 2005).

Evidence of interventions that address well-being and burnout specific to the critical care health professional community is lacking. In an umbrella review and realist-theory synthesis (in press) that focused on individual-level interventions, we identified contextual factors and mechanisms of interventions that broadly applied to the healthcare workforce (Adnan et al., 2022). While we were able to determine what interventions were likely to work, for whom and under what circumstances in the general population of healthcare professionals, our theoretical synthesis and preposition were not specific to the unique needs and profile of CCHP (because of a limited number of original intervention studies in CCHP). Additionally, the umbrella review identified gaps in understanding how self-efficacy, self-care, social support, and awareness/mindfulness may improve emotional intelligence and resilience—well-being indicators (Adnan et al., 2022). Moreover, there was insufficient evidence to consider the context-mechanism-outcome configurations for the critical care population. To address the need for and support the design of targeted interventions to CCHP that are theoretically sound (thus likely effective) and feasible, this study sought opinions from critical care experts that determined contextual factors and mechanisms of potential interventions.

## Objectives of this review

This study’s aim was to gather expert opinion to determine what types of individual-focused interventions work, under what circumstances and how to improve well-being and decrease burnout for Critical Care Healthcare Professionals (CCHP). The specific study objectives were to:

1. Gather expert opinions on the anticipated effectiveness of implementing one or a combination of individual-focused interventions to improve well-being and decrease burnout among CCHP.
2. Identify issues relating to the feasibility of implementing identified singular or combination of intervention(s) among CCHP.
3. Identify and confirm uncovered and present ideas about mechanisms and contextual factors that may contribute to the effective (or ineffective) implementation of singular or combination intervention(s).

## Materials and methods

This prospective qualitative study used semi-structured interviews with a realist evaluation method. In addition, this study was reported in adherence to the Realist And Meta-narrative Evidence Synthesis II: Evolving Standards (RAMESES II) guideline (Wong et al., 2016). This study received ethics approval from Flinders University Human Research Ethics Committee.

### Program theory

The paper used our earlier umbrella review’s program theory to understand the context, mechanism and outcome of individual interventions aimed at improving well-being and decreasing burnout (Adnan et al., 2022). The main elements of the program theory are described in Supplementary File 1.

### Study population

A total of 21 critical care healthcare professionals were included in the advisory interviews. The critical care healthcare professionals included the following eight professions: intensivist, registered nurse, psychiatrist, social worker, speech pathologist, physiotherapist, psychologist, and dietitian, as summarized in Table 1. The authors used the World Health Organization’s definition of healthcare professionals (World Health Organization, 2019) and the American Association of Critical-Care Nurses definition of “critical care” (Every Nurse, 2018). These definitions are presented in Supplementary File 2.

**Table 1:** Demographics and professional characteristics pf participant’s current practice.

Characteristics	*n* (%) of participants
**Response rate**	
Invited and participated	21 (100%)
**Gender**	
Male	6 (29%)
Female	15 (71%)
**Role in critical care**	
Senior registered nurse	8 (38%)
Intensivist	3 (14%)
Psychiatrists	2 (10%)
Speech pathologist	2 (10%)
CC clinical psychologist	2 (10%)
Senior medical registrar	1 (5%)
Social worker	1 (5%)
Physiotherapist	1 (5%)
Dietitian	1 (5%)
**Area of specialty**	
Clinical	15 (71%)
Academia	6 (29%)
**Country/state of service**	
South Australia	7 (33%)
New South Wales	4 (19%)
Queensland	4 (19%)
New Zealand	3 (14%)
Western Australia	1 (5%)
Victoria	1 (5%)
Australian Capital Territory	1 (5%)

CC, critical care. Percentages (%) depicted in this table have been rounded to full number.

### Experts

This expert opinion defined “experts” using Grundmann’s (2017) definition, which included individuals that possessed experience, technical skills (including intellectual and manual skills), judgment, trustworthiness, knowledge dissemination, and the ability to provide advice to others (Grundmann, 2017). Notably, technical skills are not merely knowledge; they include comprehensive reflections on relevant science and scientific activity (Grundmann, 2017). Therefore, in this paper, participants were screened based on these criteria to be included in the interview process.

### Recruitment

The authors invited relevant organizations in Australia and New Zealand (Australian and New Zealand Intensive Care Society) to contribute toward the expert panel process by distributing a flyer and supporting the nomination of representative expert individuals from the critical care workforce. Key experts through professional networks were also identified, directly emailed, and invited to participate. The authors ensured maximum variation in sampling to reflect diversity in terms of profession, professional experience, and skill level. The information sheet, pre-readings, and interview time and day were sent and arranged prior to the interviews. The information sheet included information about the preparation required for the interview and completion of the pre-reading of the preliminary results of the umbrella review in the form of Context-Mechanism-Outcome Configuration (CMOC) results tables.

The CMOC tables (Supplementary File 3) were provided (in confidence) with a relevant selection of provisional results of the umbrella review (in press), being a modified version of the results table that described the intervention’s context (i.e., population and setting), mechanism (i.e., how/why the intervention works, duration, context, follow-up), and outcomes (i.e., implicit and explicit reasoning and effectiveness) (Adnan et al., 2022).

### Semi-structured interviews

Each semi-structured interview consisted of up to 16 questions and began with context-setting before the in-depth and core questions (DeJonckheere and Vaughn, 2019). An interview guide was developed and used, which had been pilot tested (research assistant, NA) in three mock interviews (two clinical psychologists, one senior registered nurse expert) to ensure ease of comprehension, validity (able to extract appropriate data that correlated with the aim), and guidance in the pace of the interview (i.e., not to exceed the time limit of 30 min per interview; DeJonckheere and Vaughn, 2019). Three questions (to the initially proposed 13 questions) were added as a result of the pilot interviews, which was directed to only clinical psychologists. The questions sought to determine the validity of each intervention’s proposed contextual factors and reasonings. All authors agreed upon all changes made to the guiding questions to form the final interview guide. This was part of ensuring questions were open-ended where appropriate, clear and neutral, and avoided leading language and jargon unsuited to this professional but multi-disciplinary group of participants (DeJonckheere and Vaughn, 2019). The 13 questions asked of all participants were about the perceived effectiveness of interventions (outcome) and reasoning (context and mechanisms; Supplementary File 4).

Interviews were conducted in real time *via* an online meeting platform (Zoom Video Communications, I, 2021). The first interviewer (research assistant) asked experts a range of questions. The lead researcher (NA) was additionally present during each interview, with the role of taking observational notes, making reflective memos and answering any direct questions from the interviewer or participant that sought clarification of information and only if required to ensure the smooth continuation of the interview and depth of opinion obtained (NA). Observational notes and reflective memos were recorded in a pre-defined form to ensure consistency in the note-taking process.

### Methodological rigor

To establish trustworthiness in this expert opinion, the study authors applied Lincoln and Guba’s Four-Dimensions Criteria, which included the following stringent criteria: credibility, dependability, confirmability, and transferability (Lincoln and Guba, 1986). These criteria were chosen because of the commonality of their prior use in other contexts of qualitative health research, their practical process to meet the trustworthiness criteria for qualitative processes, and their applicability to expert opinion methodology where there is a need to minimize bias (Supplementary File 5; Lincoln and Guba, 1986; Nowell et al., 2017). Furthermore, the semi-structured interviews were carefully planned and conducted based on the four-dimension criteria to assess and ensure the robustness of the expert opinions.

### Data extraction and synthesis

Expert opinions were evaluated using an interpretative evaluation strategy where notes recorded by authors during interviews were accumulated and re-checked with the recording in order not to lose information and avoid any corresponding distortion. The main statements by experts were checked independently by two authors (NA and DC) and respective notes (Korber and Becker, 2017). Each interview was also discussed (NA and DC) regarding its contribution to the Context-Mechanism-Outcome Configuration (CMOC). In the context of this study, the authors did not record non-verbal elements (Korber and Becker, 2017). Preliminary codes were initially generated, and overarching themes were identified from these codes. Within each theme, data were categorized into context, mechanism, and outcome configurations (Supplementary File 6) and examined for: *what individual interventions are effective and ineffective (outcome) when implemented on CCHCP (context)? What causes these effects (mechanisms), and what internal and external influences (context) produced this outcome?* Pawson and Tilley (1997) defined *context* as the condition in which the intervention is being introduced, ensuring relevance to the operation of the mechanism (i.e., demographics, relationships, technology; Pawson and Tilley, 1997; Salter and Kothari, 2014). *Mechanism* describes the underlying processes and how the intervention may produce the *outcome* (Salter and Kothari, 2014).

Data (empirical evidence) were synthesized by connecting underlying causal processes to Context-Mechanism-Outcome Configurations (CMOC) in order to produce theory prepositions (Shearn et al., 2017). To determine causal processes, authors initially employed abductive and retroductive logic of internal relations of a phenomenon (i.e., intervention) to facilitate abstraction (Meyer and Lunnay, 2013). Both abductive and retroductive inferences are analysis tools that were used to refine and redevelop a conceptual framework or theory (Meyer and Lunnay, 2013). Retroduction sought to identify sufficient and necessary conditions and causes for the phenomenon to exist (Armet, 2013). Moving between data and theory, abductive reasoning was also used to compare, explore, and explain observable patterns within the data while exploring non-observable data that was overlooked by the umbrella review’s program theory (Armet, 2013). Next, the authors used information from both retroduction and abduction to create associations (iteratively hypothesizing how an outcome is achieved using identified mechanisms) and recontextualize data (determining the context within which those mechanisms were triggered; Armet, 2013). Authors (NA and DC) discussed potential explanations, strategies, and new findings to refine CMOC, which then facilitated the creation of new plausible conclusions (Armet, 2013). Authors (NA and DC) then conceptualized the necessary processes and generative mechanisms used to create empirical outcomes (Armet, 2013). Next, abstract conceptualization was used, which involved learning about the phenomenon using theories, ideas, and logic to understand the phenomenon (Armet, 2013). An empirical social product was created, followed by the generation of a conceptual map and evaluation of claims for causality using realist criteria to create theory prepositions (Armet, 2013). Next, the authors (NA and DC) determined the relationships and connections between theory prepositions, which created a web of causation that reflected a rich picture of the process and integrated into the umbrella review’s program theory to enable refinement (Adnan et al., 2022). The refined program theory is a full collation of supporting evidence.

## Results

### Demographical and professional characteristics of participants

A total of 21 critical care experts were invited and individually interviewed. There were *n* = 15 female and *n* = 6 male participants, with *n* = 18 from Australia and *n* = 3 from New Zealand. A summary of the demographical and professional characteristics of participants is demonstrated in Table 1.

### Context-mechanism-outcome configuration

Four overlapping theoretical prepositions were generated explaining *what supported successful implementation and uptake of interventions among critical care healthcare professionals*. Four middle-range theories were used during data synthesis, which guided the development of theory prepositions: (1) skill acquisition theory (VanPatten and Williams, 2014), (2) self-determination theory (Patrick and Williams, 2012), (3) social capital theory (Machalek and Martin, 2015), and (4) collaboration theory (Hurwitz and Adair, 2014). Descriptions of these theories in conjunction with the presented data/theory prepositions are provided in Supplementary File 7. Theory Prepositions (TP) developed within the data synthesis are described in Table 2. To promote transparency, the data described below also provided memo notes taken by the second interviewer (NA).

**Table 2:** Theory preposition (TP) using context-mechanism-outcome configuration (CMOC).

Identifier	Theory prepositions using context-mechanism-outcome configuration
TP1	Interventions that promoted knowledge and skill development (personal growth initiatives) *(C)*, facilitated self-awareness *(M)* enabling individuals to exercise self-regulation with the assistance of appropriate resources *(O)*.
TP2	Critical care healthcare professionals assessed and established the intervention’s effectiveness using evidence-based knowledge—where the ability to justify facilitated ease in the translational process of the intervention. Having full awareness on its credibility *(C)* facilitated autonomy in their assessment and judgment of the intervention *(M)*. This led to successful implementation of the intervention, which may allow prolonged usage of the intervention *(O)*.
TP3	Interventions that were easily accessible and inclusive *(C)* provided opportunities for critical care healthcare professionals to interact with the resource *(M)*, which facilitated feelings of acceptance and enabled a larger reach to different social groups (i.e., organizational, cultural, personality barriers) *(O)*.
TP4	Interventions should be co-produced *(C)* as it facilitated collaboration *(M)*, which meets end-users’ expectations and needs *(O)*.

C, Context; M, Mechanisms; O, Outcomes.

#### Theory preposition 1 (TP1): Interventions focused on knowledge and skill development to enhance self-regulation

Knowledge and skill development was identified to be a common theme among interventions (advocated as effective by experts). For example, resilience training, mindfulness, cognitive-based intervention, and communication and stress management skills promoted education and learning for self-improvement. One expert *(Critical Care Clinical Dietitian, 7 years of experience)* described having conflicting preferences when choosing the most effective intervention for critical care healthcare professionals. However, the expert concluded that all interventions required education and skill development.

*Everyone may choose an intervention based on their priorities and how the intervention may help them. It is normal because everyone is different, but in the end, many of the interventions require education so that skills can be developed and used.*

Another expert *(Intensivist, 9 years of experience as a specialist)* supported the idea of interventions that comprised of education as its foundations.

*Using an intervention will allow us to be educated about an issue, which can be used to improve our day-to-day encounters. Education enables us to reason why we need to know something rather than looking at it as just faith. Without educating ourselves or improving our knowledge and skills, we will not be able to advance and overcome stressful and challenging experiences.*

Self-awareness interventions also positively influenced the ability of critical care healthcare professionals to acquire personal development skills. For example, an expert suggested that mindfulness (a type of self-awareness intervention) facilitated positive perceptions of a stressor by putting situations into perspective, resulting in positive reactions. Similarly, debriefing interventions facilitated knowledge and skill development by sharing new knowledge through discussions, collaborations, and reflection. Shared information is learnt, used, and implemented in future stressful situations. The expert below *(Intensivist, 15 years of experience)* reflected on the debriefing process, which led to the uptake of emotional intelligence skills. Thus, this can be used within the workplace to overcome stressors.

*Team debriefing or team discussions can normalise an experience, and it helps to de-escalate the issue in one’s mind. This way, we can rationally think about our circumstances and use appropriate resources.*

The ability to become emotionally aware and self-regulated in challenging situations is promising, as experts noted potential opportunities for strengthening an already resilient cohort (critical care healthcare professionals).

#### Theory preposition 2 (TP2): Justifying interventions using evidence-based knowledge

Experts reported that critical care healthcare professionals required evidence to rationalize how and why an intervention is effective. The iterative requirements of ensuring that clinical interventions and performances are evidence-based have shaped their daily thought process to ensure the credibility of information before implementing it within their daily practices. Critical care health professionals use evidence-based reasoning daily within their workplace. Consequently, any intervention implemented needed to be based on similar rigor before being accepted by this group, as stated by an expert *(Critical Care Senior Registered Nurse, 28 years of experience).*

*These interventions work because they are evidence-based; this is how critical care healthcare professionals work. It is a cause-and-effect response…we are medically minded, that is, using evidence and education – what is the practice and its benefits.*

Experts suggested that interventions such as resilience training, communication and stress management, and mindfulness and cognitive interventions were “logical” (provided sound reasoning to their effectiveness) and would resonate with critical care healthcare professionals. One expert *(Intensivist, 18 years of experience)* suggested that these interventions were proposed as “logical” due to the abundance of literature and evidence of their effectiveness.

*These interventions would work within the critical care workforce because there is a lot of research and evidence to prove their effectiveness. I have also seen the effects of these interventions within the clinical setting, and there are positive results in terms of improved wellbeing and decreased stress.*

#### Theory preposition 3 (TP3): Accessibility and inclusivity of interventions

Nine experts reported that implementing the intervention would be practical and effective in terms of consistency and long-term use. One respondent *(Critical Care Senior Registered Nurse, 22 years of experience)* explained that interventions should be implemented within a whole system as factors that facilitated well-being and burnout are interrelated. For example, implementing communication skills on an individual level does not prevent the employee from being affected by negative conversations by their colleagues.

*It is not only about the individual but rather a whole system. If you instil changes within the system, then everyone will follow.*

Another expert *(Critical Care Senior Registered Nurse, 28 years of experience)* suggested that exhaustion may also influence the accessibility to interventions.

*Exhaustion potentially limits the critical care workforce to use an intervention. If you have an intervention done during personal time, it becomes hard to think about it and do it. It would be better to have the intervention during your work hours and within the clinical setting.*

Twelve experts supported interventions used during the critical care healthcare professional’s time. This decision was majorly influenced by workplace factors such as negative work cultures and lack of management support—preventing accessibility and engagement to interventions as mentioned by one expert *(Intensivist, 9 years of experience as a specialist)*.

*If you are in a unit that allows you to show your vulnerability and talk about wellbeing, then it can be an excellent place to implement the intervention (i.e., debriefing) – vice versa, it would be difficult to implement depending on the workplace culture.*

Other experts have also emphasized the extreme work environments within critical care settings. One expert suggested the following *(Intensivist, 9 years of experience as a specialist).*

*Expecting staff to engage in interventions can be difficult if they are working hard already.*

Other experts justified that implementing interventions at an individual level should not hinder the accessibility and consistency in using the interventions. One expert stated the following *(Critical Care Senior Registered Nurse, 10 years of experience)*.

*Critical care nurses would be motivated to participate in something good for them…they became nurses for a reason…don’t think that they will leave their role unless they are unable continue due to stress and burnout.*

One expert proposed using digital technology to ease accessibility, for example, using online debriefing sessions or mobile applications for mindfulness practice.

#### Theory preposition 4 (TP4): Collaboration using co-production

Interventions enforced by managers tended to be short lasting and created an environment of reluctance. Experts reported that a top-down approach would be ineffective as critical care healthcare professionals felt unvalued of their feelings, thoughts, and ideas. As an expert *(Critical Care Senior Registered Nurse, 22 years of experience)* stated.

*Ask people what they would like (implemented). People will provide suggestions and feel valued and invested in the project, which means that they will be more likely to implement it (intervention)… when imposed, people become less motivated.*

Instead, working with critical care healthcare professionals and co-producing interventions was reported as potentially effective as it allowed end-users’ expectations to be met. This process also considered differences in individual needs, which can be more engaging according to an expert *(Critical Care Senior Registered Nurse, 22 years of experience)*:

*People will resonate with different interventions, and everyone is at different levels as an individual, so it might be more beneficial to have interventions that addressed issues that the person may want to improve.*

#### Other established theory prepositions

This study identified theory prepositions that were previously reported in the umbrella review (Adnan et al., 2022). This included (1) the use of a tailored intervention, (2) the process of learning and education, (3) engagement, and (4) maintaining the quality of interventions in terms of delivery, duration, and intensity. Due to the similarity of these findings, the authors have decided not to repeat the reporting of these findings. Nevertheless, these theory prepositions demonstrated similarities between critical care healthcare professionals and general healthcare professionals.

## Discussion

Twenty-one experts were interviewed to determine what individual interventions work for the critical care workforce, under what circumstances, and why. The program theory from an umbrella review was used to guide the questions and develop a refined program theory exclusively directed to critical care healthcare professionals (Adnan et al., 2022). A wide range of experts from differing professional fields was included within the population sample, encompassing in gender, state/country, and specialty (academic/clinical) variations. As a result, the depth and breadth of the population reached theoretical saturation and met this study’s objectives. The interviews extrapolated four theory prepositions, which led to the identification of five main themes discussed below.

### Personal growth and self-awareness

*Personal growth initiatives* were a prominent theme within interventions endorsed by experts. This is referred to as attaining skills for self-improvement by using cognitive skills (planfulness and readiness) and behavioral skills (utilize resources, intentional behavior; van Woerkom and Meyers, 2019). High levels of personal growth protect individuals from psychological distress (van Woerkom and Meyers, 2019), which mirrors the effects of (expert endorsed effective) interventions – such as debriefing, mindfulness, and cognitive behavioral therapy. For example, mindfulness interventions develop the capacity to accept and tolerate painful experiences (personal growth and development) by acquiring resources that help stabilize distressful effects and reduce impulsivity (Segall, 2005). van Woerkom and Meyers (2019) suggested that interventions surrounding personal growth and development restructured perceptions of stressors to build and enhance self-confidence, which provides opportunities for positive changes and growth (van Woerkom and Meyers, 2019). The concept of self-awareness resonates with the ideologies of personal growth and development (Sutton, 2016). Self-awareness enable individuals to perceive their traits, behaviors, and feelings (Drigas and Papoutsi, 2018). It facilitates positive thought changes, allowing changes in emotions and, eventually actions (Drigas and Papoutsi, 2018). Reflection is a prime example of this process, where the cycle commences with awareness, description, analysis, evaluation, learning and eventually leads to the development of new knowledge and skills (Jayatilleke and Mackie, 2013). Experts suggested that the lack of awareness and recognition of low well-being and high burnout hinders opportunities to cope with its symptoms and consequences. Self-awareness is paramount to preventing burnout—also known as self-knowledge (Harman, 2010). Relative to the concept of anosognosia, if individuals lack the insight or awareness of a condition, in this case, burnout, it can precipitate undesirable behaviors such as misperceptions, conflicts, recklessness, and avoidance of treatments (National Alliance on Mental Illness, 2022). Likewise, if individuals are not self-aware of their burnout experience, they may not be interested in utilizing interventions to overcome their negative experiences (National Alliance on Mental Illness, 2022), impacting the usability and sustainability of interventions.

Reflection such as reflective writing, meditation, and debriefing, are effective in developing self-awareness and overall improvement of burnout (Harman, 2010). When investigating the transactional model of burnout, it is prominent that all three stages, including (1) job stressors, (2) individual strain and (3) defensive coping, are relative to the concept of self-awareness (Maslach and Leiter, 2016). That is, in the component of (1) job stressors, the Job-Demands-Resources (JD-R) model proposed that an imbalance between work demands and individual resources may contribute to (2) individual strain that can elicit an emotional response such as anxiety and exhaustion (Maslach and Leiter, 2016). It is theorized in the program theory (from the umbrella review) that decreased job stress awareness discouraged the use of resources; this is also relative to the concept of anosognosia and avoiding treatments (National Alliance on Mental Illness, 2022). Consequently, the lack of awareness and non-use of resources led to an automatic (3) defensive coping behavior and attitude such as increased cynicism, a component prominent within the gold-standard definition of burnout (Maslach and Leiter, 2016).

### Evidence and credibility

A prominent opinion among experts included the notions that attributes of rationality and evidence-based knowledge in decision-making processes are often found among critical care healthcare professionals. Rationale decision-making is defined as a multi-step process that favors objectivity, logic, and analysis over insight and subjectivity (Prowle, 2020). Rationale behaviors are often referred to as a decision-making process that chooses the optimal level of utility or benefit for the individual (Lighthall and Vazquez-Guillamet, 2015). Although there were no identified studies that supported explicitly or opposed such claims, it is evident that there is a growing recognition of the applicability of intuitive strategies such as the heuristic and pattern recognition to be applicable within high acuity environments such as among critical care practitioners (Lighthall and Vazquez-Guillamet, 2015). The critical care workforce environment is recognized for its highly stressful and uncertain environment (Piquette et al., 2009). Healthcare professionals in this environment care for patients with few physiological reserves; hence, such professionals are forced to utilize and make timely and accurate decisions (Lighthall and Vazquez-Guillamet, 2015). Often, such decision-making is based on both the hypothetical-deductive model and intuitive methods, creating a hypothetical framework for subsequent data collection and analysis and subjective experience and recognizing clinical patterns, respectively (Lighthall and Vazquez-Guillamet, 2015). Rationality falls into the intuitive method of thinking, where authors such as Djulbegovic et al. (2018) explained that in evidence-based medicine, actions and beliefs are justified by evidence trustworthiness and reliabilism (Djulbegovic et al., 2018). Therefore, if the evidence is of higher quality, calibrating the estimates of harms and benefits are enhanced (Djulbegovic et al., 2018) due to the premise that “rational people respect theory evidence” (Djulbegovic et al., 2009). This relates back to the results, where experts suggested that even though the proposed interventions provided logical and robust evidence on why or why not an intervention may work, the explanation of interventions lacked a depth of evidence and the exploration of physiological mechanisms. Thus, experts suggested that critical care health professionals would be captivated by interventions with strong logical evidence as it provided credibility and coincides with their thinking process. By providing solid evidence on the effectiveness of interventions, it provides strengths of evidence to appropriately consider the application of intervention within one’s life through the core principles of rationality—provides the autonomy to consider components such as benefits, harms, goals, reliability, probabilities, uncertainties, context, constraints, and ethics and morality (Djulbegovic et al., 2018).

### Autonomy in assessment and judgment

**Figure 1:**
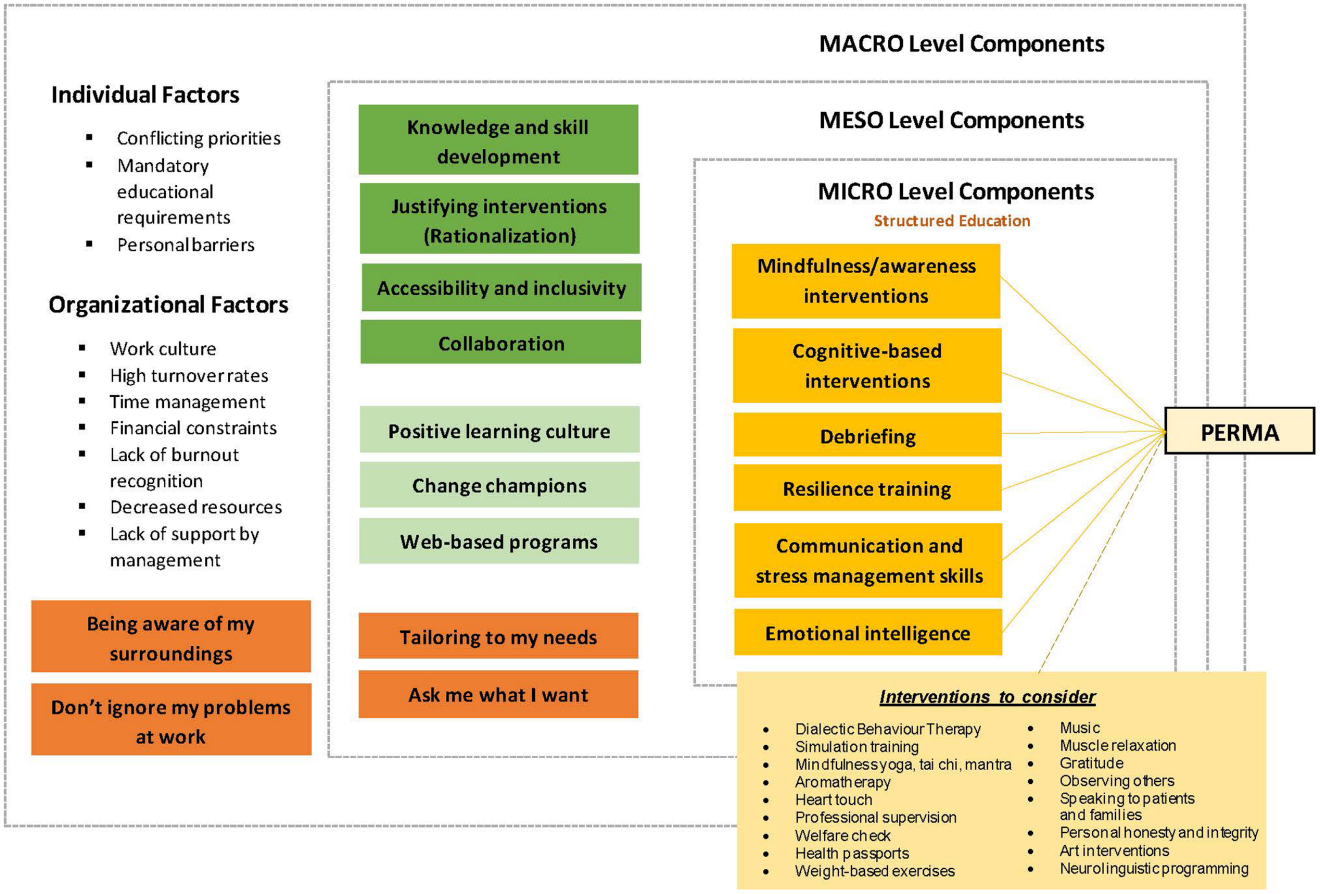
Revised program theory.

The ability to provide evidence also facilitate the autonomous decision process of utilizing an intervention. Autonomy is a critical concept proposed both by experts and within literature (Wancata and Hinshaw, 2016; Wensing and Grol, 2019). Experts suggested that if interventions are enforced on the individual by higher authority, it is likely that interventions would not flourish and produce ideal well-being outcomes. Wancata and Hinshaw (2016) proposed that respecting an individual’s autonomy enable them to make the best decision for themselves, as they are the best judges of those interests (Wancata and Hinshaw, 2016). For example, a physician does not decide for their patient even though they possess a depth of knowledge that the patient may not have. Instead, the physician may guide the patient through the process (Wancata and Hinshaw, 2016). Likewise, both experts and literature such as Wensing and Grol (2019) and Miech et al. (2018) suggested that a key stakeholder or a change champion should “guide” the process of intervention uptake to enable successful implementation and utilization of the intervention (Miech et al., 2018; Wensing and Grol, 2019). The revised program theory is demonstrated in Figure 1, and its summary is located in Supplementary File 8.

### Accessibility

Experts suggested that the component of accessibility to interventions is paramount to enable successful uptake. A combination of organizational and personal barriers impeded successful access and uptake of the intervention. Experts suggested that modifying the intervention to suit the individual’s needs may be promising in minimizing such barriers. For example, integrating web-based instruments such as video conferencing and applications. Hersch et al. (2016) conducted a study using seven web-based intervention modules consisting of nurses sending emails describing their work environment’s main stressors (Hersch et al., 2016). The nurses were then provided with directions on how to deal with the work stressor, which subsequently demonstrated overall improvements in stress management (Hersch et al., 2016). Another study that investigated the effects of web-based life skills education demonstrated improvements in burnout experiences (Yektatalab et al., 2020). Heber et al. (2017) compared the use of web- and computer-based intervention with face-to-face interventions for stress management and found no differences in the outcomes of depression or stress levels (Heber et al., 2017). However, the benefits of web-based interventions extended to their ability to have greater reach and facilitated a platform that reaches affected individuals at earlier stages of their burnout experiences (Heber et al., 2017).

### Collaboration using co-production

Collaboration was also a significant theme raised during the interview, where experts suggested that individual interventions should be created based on the needs of end users—that includes seeking their advice on what would or would not work. Research suggested that it is currently widely acknowledged that stakeholder involvement is paramount to enhancing the quality of healthcare delivery (Spanò et al., 2018). Integrating the involvement of stakeholders within the planning and development stage of projects can facilitate the successful implementation of interventions and enhance sustainability and scalability within the healthcare workforce (Lazo-Porras et al., 2020). Moreover, the shared experience of co-production may leave a more beneficial and enduring legacy compared to traditional service development – often due to service user satisfaction (Brook et al., 2020). Co-production led to interventions that are more likely to meet end-users’ needs through transparency, accountability, learning, responsiveness, and trust, all of which led to a more responsive organization (Vanleene et al., 2015). Although experts raised no limitations regarding the use of co-production of individual interventions, Flinders et al. (2016) suggested that factors such as the “rhetoric-reality gap” between the promised and delivered “co-produced” intervention was a significant impedance of successful utilization of co-production (Flinders et al., 2016). That is, despite the positive normative spirit of co-production, there exists a possibility where interventions may not meet the high expectations of end-users – often termed as an expectation gap (Flinders et al., 2016). Another significant gap in co-production included the assurances of validity, how lived experiences can be translated into academic knowledge and what information may be lost in translation (Flinders et al., 2016). This is relative to the research objectivity and results, where the interests of researchers or partners can have an effect on questions asked in the co-production stage and the inclusion of information that may be deemed interesting or valuable (Flinders et al., 2016). Although limitations exist in the concept of co-production, experts proposed that the absence of co-production can decrease the utilization and sustainability of the intervention, as observed in many confounding factors within studies.

## Strengths, limitations, and avenues for future research

This expert realist approach was able to determine the context, mechanisms, and outcome of individual interventions and understand why, how, and under what circumstances interventions may work among critical care healthcare professionals. It included a multi-disciplinary population enabling a robust representation of the nursing and medical workforce. However, it was not possible in this review to determine if questions directed to only psychologists reached theoretical saturation due to the inclusion of only two clinical psychologists. Nevertheless, expert interviews reached a point of theoretical saturation on all other questions.

As previously discussed, contextual factors such as work culture, high turnover rates, time management, and the lack of resources and support from management influenced how interventions are used within the critical care workforce. Determining the components of what makes the intervention useful and applicable for the workforce was beneficial for future applications within the “real-world.” Components such as ensuring interventions promote knowledge and skill development are evidence-based, accessible, inclusive, and take on a collaborative pathway were discussed as prime importance for critical care to accept and use the intervention. Future research, such as piloting individual interventions and integrating these theoretical findings may be promising to gain a greater understanding of its effectiveness for future translation and implementation in the “real-world” setting—potentially providing a unique evidence-based solution to improve well-being and decrease burnout among critical care healthcare professionals.

## Data availability statement

The original contributions presented in the study are included in the article/Supplementary material, further inquiries can be directed to the corresponding author.

## Ethics statement

The studies involving human participants were reviewed and approved by Flinders University Human Research Ethics Committee (ID 4901). Verbal informed consent to participate in this study was provided by the participants.

## Author contributions

NA, CB, HD, and DC: conceptualization, methodology, validation, formal analysis, visualization, and writing—review and editing. NA: writing—original draft preparation. All authors contributed to the article and approved the submitted version.

## Funding

This research was supported by the Caring Futures Institute in the context of recruiting a research assistant to conduct the interviews (seed funding; principal investigator: DC).

## Conflict of interest

The authors declare that the research was conducted in the absence of any commercial or financial relationships that could be construed as a potential conflict of interest.

## Publisher’s note

All claims expressed in this article are solely those of the authors and do not necessarily represent those of their affiliated organizations, or those of the publisher, the editors and the reviewers. Any product that may be evaluated in this article, or claim that may be made by its manufacturer, is not guaranteed or endorsed by the publisher.

## Abbreviations

CCHP: Critical care healthcare professionals
RAMESES II: Realist and Meta-narrative Evidence Synthesis Evolving Standards II
CMOC: Context-mechanism-outcome configuration
TP: Theory preposition
