# Supplementary material for: What are the essential components to implement individual-focused interventions for well-being and burnout in critical care healthcare professionals? A realist expert opinion
Source: Front Psychol. 2022 Sep 28;13:991946. doi: 10.3389/fpsyg.2022.991946 (PMC9555236; doi:10.3389/fpsyg.2022.991946)
Supplement: Supplementary file 1 [file Data_Sheet_1.PDF]

Supplementary File 1- Initial Program theory further explanation

The work environment of critical care healthcare professionals (CCHP) is a major contributor to emotional distress that leads to burnout experiences. Work environments such as working within silos and lack of a system to support collaboration between CCHPs are examples that contribute to feelings of isolation and amplification of negative emotional impact. Practices of awareness and mindfulness were suggested as enablers of cognitive flexibility, which facilitates resilience and improved wellbeing. Resilience is associated with protective factors, particularly emotional stability (decreasing neuroticism). In addition, improving lifestyle and self-care was also suggested as important components for CCHPs, as it prevented the use of negative coping techniques such as escape-avoidance behaviours. The use of escape-avoidance behaviours is significant amongst intensive care employees (Isa et al., 2019). Although it may provide an immediate or short-term stress relief, employees may use escapist fantasies, which often leads to the exacerbation of stress experiences (Isa et al., 2019). Having high levels of high self-efficacy and social support can also improve lifestyle and coping behaviours, especially amongst stressful professions such as the CCHP population. The interconnected components (self-care, social support, awareness/mindfulness) can thus facilitate development of emotional intelligence, and consequently, the improvement in resilience.

Supplementary File 2 – Study population definitions

Healthcare professionals was defined by the World Health Organization (World Health organization, 2008). This includes the following professions: Generalist and specialist medical practitioners, nursing, midwifery, traditional and complementary medicine professionals, paramedical, dentist, pharmacist, environmental and occupational health and hygiene, physiotherapist, dieticians and nutritionists, audiologist and speech therapist, optometrist and ophthalmic opticians, health professionals not elsewhere classified.

Critical care was defined by the American Association of Critical Care Nurses (Every Nurse, n.d.), which includes the following: Intensive care unit (adult, neonatal, and paediatrics), cardiac care units, emergency departments, cardiac theatre labs, telemetry units, step-down units, and recovery rooms.

Supplementary File 3 – Context-Mechanism-Outcome Configuration (CMOC) Tables

CMOC Table 1: Effective interventions for mindfulness training

NOTE: *Highlighted demonstrates interventions including population of critical care health professionals*

	Intervention	Citations	Context: *Macro-context*: Population, setting	Mechanism: *Micro-context* (Resources): **How/Why** the intervention work?	Duration	Content	Follow-up	Reasoning *(implicit/explicit presence of theory)*	Outcomes: Effective	Ineffective	Inconclusive
1.1	Mindfulness -based stress reduction (MBSR)	7	Healthcare professionals/ students, disability professionals, psychologists, counsellors, mental health professionals; in medical, Intensive care unit, paediatrics, psychiatry, radiology, surgery, gynaecology, multiple, unspecified setting, emergency department, medicine, primary and secondary care, NR	MBSR includes intensive mindfulness meditation (MM) and yoga practices/standard meditation program, which integrates Buddhist meditation with contemporary psychological and clinical practices. It alters emotional responding through the modification of cognitive-affective processes. Relative to the model of emotion regulation, MBSR may influence *attention deployment*; that is the ability to demonstrate cognitive control of negative rumination, orienting to spatial cue, and attention allocation and regulation.	2-16 weeks 30min – 2.5hours	☒	◙	Being attentive to one's inner behaviours and states can allow reflectively considered goals – responses that are situationally appropriate and self-endorsed. Doing so can impede habitual, overlearned, or automatized reactions. There is also evidence that directing one's attention to subjective emotional, mental, and physical experiences can facilitate healthy self-regulation.	empathy, emotional acceptance, emotional exhaustion, depersonalisation, personal accomplishment, mindfulness, awareness, anxiety, depression, stress, distress, anger, wellbeing, resilience, emotional intelligence, burnout, compassion fatigue, job satisfaction, self-compassion, serenity, quality of life	burnout, self-compassion, psychological wellbeing	Burnout, emotional contagion, resilience, burnout

	Intervention	Citations	**Context** *Macro-context* Population, setting	**Mechanism** *Micro-context* (Resources) **How/Why** the intervention work?	Duration	Content	Follow-up	Reasoning *(implicit/explicit presence of theory)*	**Outcomes** Effective	Ineffective	Inconclusive
1.2	MBSR and education	1	Healthcare professionals, students, trainees; setting NR	The delivery of MBSR in association with educating about mindfulness strategies and practices.	8 weeks (15 min) 30 min educate	☒	☒	Education is a process that produces a product. Such processes intentionally engage the capacities of individuals to imbue them with skills of reasoning, knowledge, values, social interactions, and socio-emotional awareness. Education enable growth producing creative, productive, engaged, and self-governing members of society.	Stress, mindfulness	Ø	Ø
1.3	Mindfulness -based positive behavioural support	2	Healthcare professionals, disability professionals, psychologists, counsellors, mental health professionals; setting NR	A customized mindfulness that utilized mindfulness interventions in combination with positive behavioural support. It includes a broad array of meditation in combination with a person-centred framework providing support to individuals in a high-risk situation or in an environment with challenging behaviours	7 days – 7 weeks	☒	☒	Places individuals in a pathway that facilitates personal transformation and transcendence.	anxiety, burnout, depression, distress, stress, mindfulness, awareness, distress, anger, wellbeing, resilience, emotional intelligence	burnout	empathy, compassion, emotional intelligence, regulation

	Intervention	Citations	Context<br>*Macro-context*<br>Population, setting	Mechanism<br>*Micro-context* (Resources)<br>**How**/**Why** the intervention work?	Duration	Content	Follow-up	Reasoning *(implicit/explicit presence of theory)*	Outcomes<br>Effective	Ineffective	Inconclusive
1.4	On-the-Job MBSR	1	Nurses; in ICU and critical care	Adaptive MBSR designed to address time-constraints of nurses.	30 days 5min	☒	☒	Occupational settings can provide opportunities to a more diverse populations and facilitate a more favourable climate – such as absence of "though, male" – oriented environments. Despite this, the location of delivery may either diminish or intensify contextual factors (i.e., implicit association of workplace with stress).	Stress	Ø	Ø
1.5	Experiential training module based on MBSR	1	Premedical/ medical/nursing/ psychology students, nurses;	A mindfulness skills course that used *vipassana* insight meditation – used concepts of breathing, body, emotion, and thoughts.	12 weeks	☒	☒	It journeys through the root of the body and mind and reduces the mind's tendency to delve into the past (reduces regrets) and future (lowers expectations and anxieties) – helps participants to remain in their current state to achieve mental tranquillity.	Empathy, emotional acceptance	Ø	Emotional contagion

Intervention		Citations	Context<br>*Macro-context*<br>Population, setting	Mechanism<br>*Micro-context* (Resources)<br>**How/Why** the intervention work?	Duration	Content	Follow-up	Reasoning *(implicit/explicit presence of theory)*	Outcomes<br>Effective	Ineffective	Inconclusive
1.6	Mindful curriculum	2	Healthcare professionals, disability professionals, psychologists, counsellors, mental health professionals; setting NR	A curriculum for a module or course that incorporates mindfulness and self-care strategies in participant's daily life to increase quality of life and build coping strategies for stress.	1 day – 10 months	☒	☒	Mindfulness skills and training does not necessarily aim at one psychiatric phenomenon, rather, it focuses on modifying underlying processes influencing the psychiatric disorder.	anxiety, burnout, depression, distress, stress, mindfulness, awareness, distress, anger, wellbeing, resilience, emotional intelligence	burnout	empathy, compassion, emotional intelligence, regulation
1.7	Telephone MBSR	4	Healthcare professionals/ students, disability professionals, psychologists, counsellors, mental health professionals; setting NR	Delivery of traditional MBSR through telephone to provide access to MBSR – without the need to attend in-person.	8 weeks 1.5hours	☒	☒	Can decrease feelings of isolation and loneliness, provides individual motivation and positive psychological states. Can overcome barriers through remote communication.	Empathy, emotional acceptance, anxiety, burnout, depression, distress, stress, mindfulness, awareness, anger, wellbeing, resilience, emotional intelligence, depression, burnout, compassion fatigue, job satisfaction, self-compassion, serenity, quality of life	Burnout	Emotional contagion, empathy, compassion, emotional intelligence, and regulation.

Intervention		Citations	Context	Mechanism					Outcomes		
			Macro-context	*Micro-context* (Resources)							
			Population, setting	**How/Why** the intervention work?	Duration	Content	Follow-up	Reasoning *(implicit/explicit presence of theory)*	Effective	Ineffective	Inconclusive
1.8	Cognitive-behaviour stress management based on MBSR	3	Healthcare professionals/ students; setting NR	Utilises materials from MBSR and cognitive therapy consists of didactic presentation, guided and training practices in mindfulness techniques, interactive exercise, group discussions, and assigned readings and audios for home use.	5-8 weeks 2 hours	☒	☒	Attempts to influence irrational thoughts whilst identifying and changing thought patterns and behaviours.	empathy, emotional acceptance, stress, mindfulness, anxiety, depression, burnout, compassion fatigue, job satisfaction, well-being, self-compassion, serenity, quality of life	Burnout, self-compassion, psychological wellbeing	emotional contagion
1.9	Interpersonal mindfulness	4	Healthcare professionals/ students, disability professionals, psychologists, counsellors, mental health professionals; setting NR	Mindfulness during interpersonal interactions, training that enables the development of awareness of oneself and others, and qualities of nonreactive presence and nonjudgements.	6 weeks 1.5hours	☒	☒	Develop considerate listening, increased presence and attentiveness, less judgemental attitudes, increased awareness of patient's perspective, change the way people operate within the workplace, and ability to prepare for and handle difficult situations	anxiety, burnout, depression, distress, stress, mindfulness, awareness, anxiety, stress, anger, wellbeing, resilience, emotional intelligence, empathy, emotional acceptance	Burnout, self-compassion, psychological wellbeing	empathy, compassion, emotional intelligence, regulation, emotional contagion

Intervention		Citations	Context	Mechanism					Outcomes		
			Macro-context	*Micro-context* (Resources)							
			Population, setting	**How**/**Why** the intervention work?	Duration	Content	Follow-up	Reasoning *(implicit/explicit presence of theory)*	Effective	Ineffective	Inconclusive
1.10	Mindfulness -based cognitive therapy (MBCT), includes mindful therapy	9	Healthcare professionals/ students, disability professionals, psychologists, counsellors, mental health professionals; NR, in medical/ICU/ critical care, paediatrics, psychiatry, radiology, surgery, gynaecology, multiple, unspecified	"Three-minute breathing space" is the key element in MBCT. It aims to assimilate learnt formal meditation practices into the participant's everyday life. There are three steps including:<br>(1) awareness of thoughts, bodily sensations, feelings<br>(2) attention to breath<br>(3) attention to body.	8 weeks 90min-10hours	☒	◙	Enables cognitive restructuring – that is the ability to identify and challenge rigid and negative thoughts. This allows for the facilitation of resilience rather than the ability to achieve a specific outcome or solve a particular issue.	Empathy, emotional acceptance, anxiety, burnout, depression, distress, stress, mindfulness, awareness, anger, wellbeing, resilience, emotional intelligence	Burnout, self-compassion, psychological wellbeing	(occupational) stress, Emotional contagion, empathy, compassion, emotional intelligence and regulation, burnout, resilience
1.11	Web-based mindfulness programme	1	Healthcare professionals; setting NR	Includes experiential audio exercises and online applied training (lifestyle coaching and personalized progress tracking).	☒	☒	☒	Promising in this era – provides ease (i.e. due to heavy work schedules), cost effective, and less time consuming	Wellbeing	Ø	Ø

Intervention		Citations	Context	Mechanism					Outcomes		
			Macro-context	*Micro-context* (Resources)							
			Population, setting	**How/Why** the intervention work?	Duration	Content	Follow-up	Reasoning *(implicit/explicit presence of theory)*	Effective	Ineffective	Inconclusive
1.12	MBST	3	Healthcare professionals/ students, disability professionals, psychologists, counsellors, mental health professionals; setting NR	A psychological intervention that consists of five components – presence, listening, empathy, compassion, and boundary awareness. MBST differs to other mindfulness-based psychotherapies as it utilises the three domains of mindfulness, that is, present moment attention, deliberate attention, and nonreactive attention.	5-7 weeks 1 hour	☒	☒	Enables inputs to enter an individual's awareness in a way that the individual does not notice of its process.	Anxiety, burnout, depression, distress, stress, mindfulness, awareness, distress, anger, wellbeing, resilience, emotional intelligence, burnout, compassion fatigue, job satisfaction, well-being, self-compassion, serenity, quality of life	Burnout	empathy, compassion, emotional intelligence, regulation
1.13	Mindfulness -Based Professional Developmen t retreat	2	Healthcare professionals, students, trainees;	Designed by psychiatrists, collaboration with a Zen Master. It involves mindfulness exercises such as meditation and mindfulness.	9 hours – 2 days	☒	◙	Enables the cultivation of benevolent mental states, which includes compassion, loving-kindness, empathic joy, and equanimity.	Stress, mindfulness, distress	Burnout, self-compassion, psychological wellbeing	Ø

Intervention		Citations	Context	Mechanism					Outcomes		
			Macro-context	*Micro-context* (Resources)							
			Population, setting	**How/Why** the intervention work?	Duration	Content	Follow-up	Reasoning *(implicit/explicit presence of theory)*	Effective	Ineffective	Inconclusive
1.16	Mindfulness meditation	3	Nurses; ICU and critical care	Meditation is used as a tool to develop mindfulness, encouraging attitudes of non-judgemental acceptance and openness, equanimity, and stillness.	8 weeks or 8 sessions 90min	☒	◙	Repeated meditation practices can enhance body awareness, self-regulation, and develop emotion regulation skills.	Stress, mindfulness and awareness, anxiety, depression, distress, anger, wellbeing, resilience, emotional intelligence	burnout	Ø
1.14	ACT based self-management (mindfulness based)	1	Healthcare professionals, students, trainees; setting NR	ACT uses mindfulness and acceptance strategies in developing behaviour change through the improvement of psychological flexibility. Such programs use metaphors and real-life examples to increase awareness of the ‘present moment’ and decrease rumination of past deeds, worries, and future problems.	1 day – 12 weeks (30min phone consult for 3 months)	☒	☒	Does not aim to replace unhealthy psychological events with healthy events, rather, concurrent cultivation of acceptance towards an unhealthy psychological event, teaches the observation of events for what they are and not being entangled with them, and enables commitment of behaviours that support living through healthy values.	stress, mindfulness, psychological flexibility	burnout, self-compassion, psychological wellbeing	Ø

Intervention		Citations	Context	Mechanism					Outcomes		
			Macro-context	*Micro-context* (Resources)							
			Population, setting	**How**/**Why** the intervention work?	Duration	Content	Follow-up	Reasoning *(implicit/explicit presence of theory)*	Effective	Ineffective	Inconclusive
1.15	Introduction to mindfulness and mindfulness in daily life	2	Healthcare professionals/ students, disability professionals, psychologists, counsellors, mental health professionals; setting NR	MBIs constitutes of a significant homework component, either guided or unguided meditation daily home practices. Include informal practices to enable the integration of mindful awareness in the participant's everyday activities.	1 hour	☒	☒	Enables individuals to focus on reactions to stress, recognize difficult thoughts and feelings, and act with acceptance.	anxiety, burnout, depression, distress, stress, mindfulness, awareness, anger, wellbeing, resilience, emotional intelligence	Burnout	empathy, compassion, emotional intelligence, regulation
1.17	Mindfulness based group psychoeduca tion activities	1	Healthcare professionals; in primary healthcare centres.	Group-based enables the sharing of personal experiences in practicing mindfulness – learn via modelling and direct feedback.	8 weeks	☒	☑	Psychoeducation provides individuals with skills, knowledge, strength, and strategies to overcome associated impairments. Moreover, it provides significant improvements in psychiatric symptoms, cognitive insight, and functioning.	Distress	Ø	Ø

			**Context**	**Mechanism**					**Outcomes**		
			Macro-context	*Micro-context* (Resources)							
**Intervention**		**Citations**	Population, setting	**How**/**Why** the intervention work?	Duration	Content	Follow-up	Reasoning *(implicit/explicit presence of theory)*	Effective	Ineffective	Inconclusive
1.18	Mindfulness education program	2	Healthcare professionals; primary care, ICU, paediatric, ED, NR	Educates formal and informal mindfulness meditation practice that aims to train non-judgemental attitudes and attentional control components of mindfulness.	8 weeks	☒	☑	Education is a process that produces a product. Such processes intentionally engage the capacities of individuals to imbue them with skills of reasoning, knowledge, values, social interactions, and socio-emotional awareness. Education enable growth producing creative, productive, engaged.	Emotional exhaustion, depersonalization, personal accomplishment	Ø	Stress
1.19	Occupationa l Mindfulness	1	Healthcare professionals/ students, disability professionals, psychologists, counsellors, mental health professionals; setting NR	Utilizes an array of components derived from MBCT (mindfulness-based cognitive therapy), MBSR (mindfulness-based stress reduction), and positive psychology. The program is designed to be customizable for the requirements of specific workforces.	8 weeks	☒	☒	Enhances the awareness of sources and signs of stress, and positively changes attitudes, interactions, and behaviours of self-care.	mindfulness, awareness, anxiety, depression, stress, distress, anger, wellbeing, resilience, emotional intelligence	Burnout	Ø

Intervention		Citations	Context	Mechanism					Outcomes		
			Macro-context	*Micro-context* (Resources)							
			Population, setting	**How**/**Why** the intervention work?	Duration	Content	Follow-up	Reasoning *(implicit/explicit presence of theory)*	Effective	Ineffective	Inconclusive
1.20	Mind-body course	2	Healthcare professionals/ students, disability professionals, psychologists, counsellors, mental health professionals;	Learn about mind-body medicine (yoga, neuroscience, breathing techniques, meditation).	11 weeks	☒	☒	It emphasizes connections between feelings, thoughts, psychology, behaviours, and corresponding impact on overall health.	anxiety, burnout, depression, distress, stress, mindfulness, awareness, distress, anger, wellbeing, resilience, emotional intelligence	Burnout	empathy, compassion, emotional intelligence, regulation
1.21	Mindfulness in motion	1	Healthcare professionals/ students, disability professionals, psychologists, counsellors, mental health professionals;	This is a type of MBI that incorporates music, yoga, and mindfulness practices on a time-limited fashion.	8 weeks	☒	☒	It is offered in method that is less time intensive, which can be delivered in the workplace – intended for busy individuals to experience the benefits of mindfulness.	mindfulness, awareness, anxiety, depression, stress, distress, anger, wellbeing, resilience, emotional intelligence	Burnout	Ø
1.22	Mindfulness -based mentoring	2	Healthcare professionals/ students, disability professionals, psychologists, counsellors, mental health professionals; setting NR	Psychiatrist mentored (using eastern holistic mentoring process) on behavioural and psychopharmacological aspects of mindfulness. Particularly, it focused on *shunyata* (emptiness).	6-11 sessions	☒	☒	Mentors assist in the willingness of individuals to empty the mind of old patters of practices and thoughts so that the individual is fully present in each moment. This assists in the ability to accept new information, development, behaviour, and strengthening of relationships.	anxiety, burnout, depression, distress, stress, mindfulness, awareness, distress, anger, wellbeing, resilience, emotional intelligence	burnout	empathy, compassion, emotional intelligence, regulation

Intervention		Citations	Context	Mechanism					Outcomes		
			Macro-context	*Micro-context* (Resources)							
			Population, setting	**How**/**Why** the intervention work?	Duration	Content	Follow-up	Reasoning *(implicit/explicit presence of theory)*	Effective	Ineffective	Inconclusive
1.23	Promotion of acceptance in carers and teachers	2	Healthcare professionals/ students, disability professionals, psychologists, counsellors, mental health professionals;	Uses acceptance- and mindfulness-based approaches – specifications on how the intervention works was not reported.	1.5 days	☒	☒	MBI enhances awareness of current moment Acceptance-based approaches incorporate mindfulness to avoid unpleasant feeling and thoughts.	anxiety, burnout, depression, distress, stress, mindfulness, awareness, anger, wellbeing, resilience, emotional intelligence	burnout	empathy, compassion, emotional intelligence, emotional regulation
1.24	Acceptance and mindfulness workshop	2	Healthcare professionals/ students, disability professionals, psychologists, counsellors, mental health professionals; setting NR	Based on principles of ACT (acceptance and commitment therapy) and utilised group discussions, didactic teaching, metaphors, written exercises, short video presentations, practical interactive exercises.	1.5 days	☒	☒	Increases psychological acceptance and mindfulness of thoughts, sensations, feelings and reducing literal control of language and thoughts of behaviours	anxiety, burnout, depression, distress, stress, mindfulness, awareness, distress, anger, wellbeing, resilience, emotional intelligence	burnout	empathy, compassion, emotional intelligence, emotional regulation

Abbreviations: NR=not reported, min=Minutes, (-)=to, ☒=Not reported in citations, ☑=Reported in all citations, ◙=reported in some citations (inconsistencies), Ø= Nil, ICU=Intensive Care unit, ED=Emergency department, MBSR=Mindfulness Based Stress Reduction, ACT=Acceptance and Commitment Therapy, MBI=Mindfulness Based Interventions.

CMOC Table 2: Other effective interventions for wellbeing

NOTE*: *Highlighted* ▭ *demonstrates interventions with population including critical care health professionals

	Intervention	Citations	Context	Mechanism					Outcomes		
			Macro-context	*Micro-context* (Resources)				Reasoning *(implicit/explicit presence of theory)*			
			Population, setting	**How/why** the intervention works	Duration	Content	Follow-up		Effective	Ineffective	Inconclusive
Cognitive-behavioural skills training											
2.1	Emotional intelligence	1	Nurses; ICU and critical care	Education through general conference programme, inter-group sessions, and educational booklets.	4 sessions 2 hours	☒	☒	Includes four aspects including (1) ability to perceive, assess, express emotions accurately, (2) use emotions to promote thinking, (3) Understand emotions and emotional knowledge, (4) ability to manage and regulate actions.	Occupational stress	Ø	Ø
2.2	Emotion regulation training	1	Nurses; ICU and critical care	Develop the ability to manage and respond to one's own emotions. There were descriptions of how the intervention works.	8 sessions 2 hours	☒	☒	Ability to break the *vicious emotional* cycle through emotion dysregulation.	Occupational stress	Ø	Ø
2.3	Education on Cognitive Behaviour Therapy (CBT) skills	1	Physicians; setting NR	Education was provided to participants, which focused on CBT skills including cognitive restricting and relaxation.	7 sessions 6 mins	☒	☑	Enable symptom reduction, improved function, and remission of disorder, individuals are exposed to problem-solving processes aimed to challenge maladaptive cognitions and modify such behavioural patterns	Occupational stress	Ø	Ø

	Intervention	Citations	Context	Mechanism					Outcomes		
			Macro-context	*Micro-context* (Resources)							
			Population, setting	**How**/**why** the intervention works	Duration	Content	Follow-up	Reasoning *(implicit/explicit presence of theory)*	Effective	Ineffective	Inconclusive
2.4	CBT – not based on MBSR	3	Healthcare professionals; primary healthcare centres, NR	Utilised emotion-focused coping to regulate the stressful emotion reaction associated with the environment by solving problems and changing negative thoughts and behaviours.	5 weeks 5 hours	☒	◙	To enable symptom reduction, improved function, and remission of disorder, individuals are exposed to problem-solving processes aimed to challenge maladaptive cognitions and modify such behavioural patterns.	Distress, emotional exhaustion, depersonalisation, personal accomplishment	Ø	Ø
2.5	Balint group	1	Physicians; setting NR	Sessions providing understanding on interaction with patients.	4-9 session s (1-1.5 hours)	☒	☒	Balint groups allows individuals to emotionally mobilize negative experiences and prevents oneself to seep deeply into personal life.	Occupational stress	Ø	Ø

	Intervention	Citations	Context	Mechanism					Outcomes		
			Macro-context	*Micro-context* (Resources)							
			Population, setting	**How**/**why** the intervention works	Duration	Content	Follow-up	Reasoning *(implicit/explicit presence of theory)*	Effective	Ineffective	Inconclusive
2.6	Resilience training	2	Healthcare professionals/ students, disability professionals, psychologists, counsellors, mental health professionals; ICU and critical care, NR	Resilience training can be summarized into three dimensions, that is understanding resilience and characteristics resilient people possesses, methods in developing resilience, and internal and external support factors. Training included question and answer, lectures, and group discussions. Delivered by trained researchers supervised by psychiatrists.	5 sessions for 8-12 weeks 90 – 120 min	☒	☒	Resilience training is 'preventative' (forward looking) in nature and focuses on fostering personal qualities required to deal with unanticipated stressors.	Occupational stress mindfulness awareness, anxiety, depression, stress, distress, anger, wellbeing, resilience, emotional intelligence	Burnout	Stress
2.7	SMART program	3	Physicians, psychiatrists; in medical/ICU/ critical care, multiple primary and secondary care, some studies unspecified	Administered as an individual training program that integrated resilience and stress management through paced breathing meditation.	Once off 90 min; 30min follow up phone call	☑	☒	Considering time constraints, SMART program can be learned in one to two brief sessions, which does not require an elaborate sitting practice.	Occupational stress	Ø	Burnout, resilience, burnout

	Intervention	Citations	Context: *Macro-context* — Population, setting	Mechanism: *Micro-context* (Resources) — **How/why** the intervention works	Duration	Content	Follow-up	Reasoning *(implicit/explicit presence of theory)*	Outcomes: Effective	Ineffective	Inconclusive
2.8	Cognitive coping strategies and problem-solving method	1	Healthcare professionals; NR	Coping training was administered as a structured education programme framework, which utilised problem- and emotion- focused coping methods.	7 weeks	☒	☑	Cognitive coping strategies assists in regulating emotions in response to a stressor. Together with problem-solving methods, it can facilitate recognition, acceptance, and solving existing problems by concentrating on one's strengths and weaknesses.	emotional exhaustion, depersonalisation, personal accomplishment	Ø	Ø
2.9	Counselling session	1	Physicians, psychiatrists; surgery, medicine, primary and secondary care, NR	Provides support, relief, and psychological first aid to individuals who are unable to cope with current situations.	1 day 6-7 hours **1 to 1**	☒	☑	Counselling programme aimed to motivate reflection of current situation and personal needs.	Occupational stress	Ø	Resilience, burnout
					5-days to 9 months 1-hour group	☒	☑		Occupational stress	Ø	Resilience, burnout
2.10	Educational workshop/seminars	1	Physicians; setting NR	Seminars focused on relaxation training, management of social-support, self-management, and practice management.	Fortnight 3 sessions 3 hours	☑	☒	Enable the sharing of evidence-based knowledge and retention of knowledge through active learning strategies and increased learning.	Occupational stress	Ø	Ø

Intervention		Citations	Context	Mechanism					Outcomes		
			Macro-context	*Micro-context* (Resources)				Reasoning *(implicit/explicit presence of theory)*			
			Population, setting	**How/why** the intervention works	Duration	Content	Follow-up		Effective	Ineffective	Inconclusive
2.11	Self-compassion intervention	1	Healthcare professionals, students, trainees; setting NR	Ability to treat oneself compassionately during personal suffering. Intervention included educating individuals to notice their own suffering, provision of informal self-compassion techniques, and applying these techniques within their daily life.	3-weeks 1.5 hours	☒	☒	Self-compassion facilitates resilience through the moderation of individual's reactions towards a negative event.	Mindfulness, self-compassion	Ø	Stress
2.12	Communication and/or stress management skills class	3	Healthcare professionals; primary care, ICU, paediatric, ED	Offered theoretical information regarding communication skills within two to three- person interviews. Theoretical sessions were facilitated via role-plays and immediate feedback by experienced facilitators. Stress management training focused on cognitive restructuring, relaxation techniques, time management, and detection of job stressor.	3 - 8 weeks 2-4 hours	☒	☒	Communication skills are acquired through learning. One component of life-skills includes stress management skills, which are paramount to the enhancement of positive psychological states, particularly in vitality and mental wellbeing.	Occupational stress, emotional exhaustion, depersonalisation, personal accomplishment	Ø	Stress
2.13	Supervised yoga instructions/ yoga nidra	1	Healthcare professionals, nursing aides and assistant, no specification; setting NR	Aims to increase brain-wave activity and creates neuroplastic effects that improves memory, mood, cognition, and anxiety.	9-12 hours	☒	◙	Allows positive influences on the quality of sleep and can also compensate for the lack of sleep.	Stress	Ø	Ø

Intervention		Citations	**Context**	**Mechanism**					**Outcomes**		
			Macro-context	*Micro-context* (Resources)							
			Population, setting	**How/why** the intervention works	Duration	Content	Follow-up	Reasoning *(implicit/explicit presence of theory)*	Effective	Ineffective	Inconclusive
2.14	"The Basic Eight" Qigong	1	Healthcare professionals, nursing aides and assistant, no specification; setting NR	The Basic Eight consists of eight exercises to activate 14 meridians, which is often utilised by acupuncturists – large-intestine, lungs, spleen, stomach, small intestine, heat, kidney, urinary bladder, heart governor, gallbladder, triple heater, liver, conception, and the governing vessels.	12 hours	☒	☑	Disease is attributed to deficiency or access of Qi – disruption of Qi to flow to organs, systems, and structures. Thus, Qi is paramount to produce optimal health (Qigong aims to restore or maintain the natural flow and balance of Qi).	Stress	Ø	Ø
2.14	Job stress awareness, PMR, assertiveness training, time management	1	Nurses; intensive care unit (ICU) and critical care	Focused on general requirement of the job, caring for patients, relationships with co-workers and supervisors, progressive muscle relaxation, and time management.	2-3 hours	☒	☒	Opportunities to discuss problems and issues at work can enable conflict management.	Occupational stress	Ø	Ø

Intervention		Citations	Context: *Macro-context* Population, setting	Mechanism: *Micro-context* (Resources) **How/why** the intervention works	Duration	Content	Follow-up	Reasoning *(implicit/explicit presence of theory)*	Outcomes: Effective	Ineffective	Inconclusive
2.15	Self-guided online self-compassion training	1	Healthcare professionals, students, trainees; setting NR	Based on emotion regulation model of self-compassion – proposing that self-compassion can assist in the relation of emotions when encountered with a stressful event, reducing the negative impact. The online training was designed based on key elements of psychotherapy and therapeutic change.	Every week 1-2 hours	☒	☒	Enables flexibility, accessibility, and sustainability for mental health prevention. Online enable cost-effectiveness on a wide scale.	Mindfulness, self-compassion	Ø	Stress
3.7	Mailed - letter with feedback on GHQ-12 score	1	Healthcare professionals; in primary healthcare centres.	GHQ-12 with an interpretation score and self-help sheet. Administered in a newspaper style with comments regarding doctor's emotional health and emotional arousal via self-reflective writing and self-evaluation activities.	Once	☒	☒	Incorporation of Transtheoretical Model of Change, proposing five key stages – pre-contemplation, contemplation, preparation, action, and maintenance.	Distress	Ø	Ø

Abbreviations: NR=not reported, min=Minutes, (-)=to, ☒=Not reported in citations, ☑=Reported in all citations, ◙=reported in some citations (inconsistencies), Ø= Nil, ICU=Intensive Care unit, ED=Emergency department, SMART=Stress Management And Resiliency Techniques, PMR= Progressive Muscle Relaxation, GHQ-12= General Health Questionnaire-12.

CMOC Table 3 – Ineffective interventions

NOTE*: *Highlighted demonstrates interventions with critical care health professionals

	Intervention	Citations	Context	Mechanism					Outcomes		
			Macro-context	*Micro-context* (Resources)							
			Population, setting	**How/Why** the intervention works?	Duration	Content	Follow-up	Reasoning *(implicit/explicit presence of theory)*	Effective	Ineffective	Inconclusive
3.1	Neuro-linguistic programming	1	Nurses; intensive care unit (ICU) and critical care	Communication framework that uses techniques to understand and enable change in one's behaviour and thinking.	6 months 3 hours	☒	☒	Demonstrates internal representations of the world is biased depending on sensory modality (auditory, visual, olfactory, kinaesthetic, gustatory). Individual's dominant modality is signalled via various behaviours, especially through eye movements and verbal expressions.	Ø	Ø	Stress
3.2	Heart touch technique	1	Healthcare professionals; primary care, ICU, paediatric, ED, NR	A method used to intentionally change thoughts and feelings. It comprises of three steps – heart centred awareness, loving touch, and connection with the higher power.	☒	☒	☒	Develops meaningful connection/commitment with other individuals by enabling feelings of love or appreciation, social support, and spirituality. Improve physical, menta, emotional, and spiritual health and wellbeing.	Ø	Ø	Stress
3.3	Career identity training	1	Healthcare professionals; primary care, ICU, paediatric, ED, NR	Assists individuals with defining their roles and how they are expected to act in a changing career context.	☒	☒	☒	Offers a more flexible base for the construction of identity.	Ø	Ø	Stress

			Context	Mechanism					Outcomes		
			Macro-context	*Micro-context* (Resources)							
Intervention		Citations	Population, setting	**How/Why** the intervention works?	Duration	Content	Follow-up	Reasoning *(implicit/explicit presence of theory)*	Effective	Ineffective	Inconclusive
3.4	Coping and support groups	2	Healthcare professionals; primary care, ICU, paediatric, ED	Coping groups integrated cognitive coping and problem-solving structured education framework. Support groups utilised the Gibbs' reflection model, which included the following components: description, feelings, evaluation, analysis, conclusion, and action plan.	6 months	☒	◙	Coping groups enable the development of problem-focused coping methods and emotion-focused coping methods – that is methods directed to change the situation and methods employed to change emotional and cognitive reactions towards a situation. Support groups enable the provision of support, information, an environment where experiences can be shared, and a sense of belonging.	Ø	Ø	Stress
3.5	Auriculotherapy	1	Healthcare professionals; primary care, ICU, paediatric, ED, NR	Stimulating specific acupoints located on the external ear. This includes acupoint acupressure, electrical stimulation, and the use of different types of needles, magnetic balls, and seeds.	☒	☒	☒	Stimulates an area of the brain, causing discharges of endorphins and the release of neurotransmitters.	Ø	Ø	Stress

Intervention		Citations	Context	Mechanism					Outcomes		
			Macro-context	*Micro-context* (Resources)							
			Population, setting	**How/Why** the intervention works?	Duration	Content	Follow-up	Reasoning *(implicit/explicit presence of theory)*	Effective	Ineffective	Inconclusive
3.6	Mental practice sessions	1	Physicians; setting NR	Systematic mental rehearsal where individuals imagine themselves performing the action without physical movements.	5 sessions 30 min	☒	☒	Enhances physical performances and cognitive skills. Responses to a negative or stressful stimulus can be moderated through visualization.	Ø	Ø	occupational stress, burnout
3.7	Debriefing sessions	1	Physicians; setting NR	Debriefing included topics on chronic work related emotional and interpersonal stressors. Includes feedback, peer support, mentoring, and problem solving.	8 weeks 1 hour	☒	☒	Provides an opportunity to meet and discuss with peers and other individuals – facilitated by a trusted senior health professional. Ability to share experiences enables potential antidotes to burnout, alongside peer-support groups that facilitates the prevention.	Ø	Ø	occupational stress, burnout
3.8	Meeting	1	Physicians, psychiatrists; surgery, medicine, primary and secondary care.	Discussion groups that discusses on elements of mindfulness, shared experiences, reflection, and a small-group learning.	3 sessions in 4 weeks 3 hours	☒	☒	This mindfulness-oriented training assists in the promotion of physician wellbeing and patient-oriented care through elements of awareness, attention, self-reflection, and intention.	Ø	Ø	resilience, burnout,

Intervention		Citations	Context	Mechanism					Outcomes		
			Macro-context	*Micro-context* (Resources)							
			Population, setting	**How/Why** the intervention works?	Duration	Content	Follow-up	Reasoning *(implicit/explicit presence of theory)*	Effective	Ineffective	Inconclusive
3.9	RISE (resilience in stressful event) intervention	1	Physicians; in medical/ICU/ critical care, paediatrics, psychiatry, radiology, surgery, gynaecology, multiple, unspecified	Peer-led psychological first aid and emotional support for individuals in a stressful patient-related or unanticipated adverse event.	☒	☒	☒	Demonstrates how to establish a peer-to-peer support program and educate multi-disciplinary teams on how to support and respond to a team member involved in a stressful situation.	Ø	Ø	Burnout, resilience
3.10	Simulation training	1		Learning objectives: surgical skills, extensive debriefing to practice diagnosis, team-based training, work-up of encountered problems, and management.	☒	☒	☒	It provides simulation-, hands-on training to improve procedural skills, medical knowledge, communication, teamwork, and professionalism	Ø	Ø	Burnout, resilience
3.11	Micro-task	1	Physicians, psychiatrists; surgery, medicine, primary and secondary care.	Crafted for physicians and designed to promote professional wellbeing and satisfaction – physicians required to choose and complete a task weekly.	10 weeks	☑	☒	Informed by six themes using literate on physical wellbeing, career satisfaction, psychology, and mindfulness. Purposefully designed to resonate with physicians in practice so that can promote positive culture, effectively implemented, and allows physicians to thrive.	Ø	Ø	resilience, burnout,

Intervention		Citations	Context	Mechanism					Outcomes		
			Macro-context	*Micro-context* (Resources)							
			Population, setting	**How/Why** the intervention works?	Duration	Content	Follow-up	Reasoning *(implicit/explicit presence of theory)*	Effective	Ineffective	Inconclusive
3.12	Adaptation practice course	1	Physicians, psychiatrists; surgery, medicine, primary and secondary care.	The course provided education on how to engage with one's moods and feelings physically rather than cognitively, and included methods of how not to engage thoughts about oneself.	6 months	☑	☒	Adaptation enables individuals to experience less stress in the same activity that was previously stressful through *overload principle* – more stress is required to be abled to the system to stimulate improvements.	Ø	Ø	resilience, burnout
3.13	Didactic or interactive instruction in biopsychosocial approach to patient care	1	Physicians; setting NR	Mixed interventions which included role play, Balint groups, and individual teaching to improve self-awareness. Workshops were provided to facilitate the interventions.	12 weeks 5-6 hours	☒	☑	Related to the concept of the biopsychosocial model that views health and illness as a product of behavioural factors, biological characteristics, and social conditions. Accordingly, the lack of self-awareness can result to negative interpretations of situations – being a challenge or a threat.	Ø	Ø	Occupation stress, burnout
3.14	Background affect trouble handling empathy (BATHE)	1	Physicians; setting NR	Instructions on BATHE psychotherapeutic tool was provided to participants, where it focused on self-empathy and awareness. Participants were then encouraged to individually practice three time a week.	3 times per week 45 min	☒	☒	Psychotherapy tools can empower feelings of trust and allow the development of positive feelings of themselves to control certain circumstances of their lives.	Ø	Burnout	Ø

	Intervention	Citations	Context	Mechanism					Outcomes		
			Macro-context	*Micro-context* (Resources)							
			Population, setting	**How/Why** the intervention works?	Duration	Content	Follow-up	Reasoning *(implicit/explicit presence of theory)*	Effective	Ineffective	Inconclusive
3.15	Relaxation response resiliency program (3RP) Palliative Care Clinicians (PCC)	1	Physicians; in medical/ICU/ critical care, paediatrics, psychiatry, radiology, surgery, gynaecology, multiple, unspecified	Based on CBT and positive psychology principles It included introduction and education facilitated by physicians from MGH (BHI).	☒	☒	☒	It elicits relaxation, reduce stress reactivity, and increase the relationship of connectedness to oneself and towards others.	Ø	Ø	Burnout, resilience
3.16	Chair Massage – offered to play music	1	Healthcare professionals; primary care, ICU, paediatric, ED, NR	Chair massage by massage therapist whilst offered preferences of music. Included, back, neck, shoulders, arms, and hands.	10 weeks 10-15min	☑	☑	Chair massage is easy to set up, can be in semiprivate areas as individuals remain clothed, and does not utilise much space.	Ø	Ø	Stress

Intervention		Citations	Context	Mechanism					Outcomes		
			Macro-context	*Micro-context* (Resources)							
			Population, setting	**How/Why** the intervention works?	Duration	Content	Follow-up	Reasoning *(implicit/explicit presence of theory)*	Effective	Ineffective	Inconclusive
3.17	Psychosocial skills training combined with cognitive behavioural and solution-focused counselling	1	Physicians, psychiatrists; surgery, medicine, primary and secondary care.	Focused on situations and problems in the 'real world', which included coping support and strategies alongside the formulation of future goals between colleagues. Training included psychoeducation (videos, oral group discussions, theoretical input, experimental exercises, self-awareness, and home assignments). Content of interventions were designed to facilitate cognitive behavioural training and solution-focused group work principles.	12 sessions 1.5hours	☒	☒	Function as a recovery-oriented psychosocial rehabilitation intervention which trains new skills and thoughts that may interfere with performances (i.e. low self-esteem) in the real world.	Ø	Ø	resilience, burnout
3.18	General Swedish Massage	2	Nurses; ICU and critical care, NR	Massage was performed on hands, legs, back, chest, lower back. Lotion were applied and massage performed using stroking motions on the surface of the skin with moderate palm pressure.	4-5 weeks 25 min	☑	☒	It aims to relax the individual through muscle manipulation – enables relaxation, stretching to relieve muscular tension or tightness, and reduces physical stress.	Ø	Ø	Stress, job-related stress

Intervention		Citations	Context	Mechanism					Outcomes		
			Macro-context	*Micro-context* (Resources)							
			Population, setting	**How/Why** the intervention works?	Duration	Content	Follow-up	Reasoning *(implicit/explicit presence of theory)*	Effective	Ineffective	Inconclusive
3.19	Respiratory One Method	1	Physicians; setting NR	Includes relaxation and meditation practices. The practitioner will repeat the word "one" or a phrase such as "let go" and individuals will then exhale.	4 session 1 hour each	☒	☒	Enhances self-regulation of one's negative emotion and thinking.	Ø	Ø	Occupational stress
3.20	Tai Chi	1	Nurses; ICU and critical care	Contents included breathing exercises tai chi practices, visualization, and cool-down exercises with assistance by a tai chi instructor with 22 years of experience.	1-2 hours	☒	☒	Emphasizes on mind-body connection. It integrates self-defence and physical strengthening using mindfulness techniques (i.e. relaxation of mind).	Ø	Ø	Stress
3.21	Inhalation	3	Healthcare professionals/ students, disability professionals, psychologists, counsellors, mental health professionals; ICU and critical care, NR	Steam infusion of therapeutic grade lavender essential oil throughout nursing units.	10-15 min for 24hr	☑	◙	There are differences in research views for inhalation therapy. Essentially, research argues that the main mechanism for aromatic therapy is between the olfactory and limbic system, where scent receptors in the nose send chemical-based messages to the brain's limbic region via the olfactory nerve.	Ø	Ø	Stress, job-related stress

Intervention		Citations	Context	Mechanism					Outcomes		
			Macro-context	*Micro-context* (Resources)							
			Population, setting	**How/Why** the intervention works?	Duration	Content	Follow-up	Reasoning *(implicit/explicit presence of theory)*	Effective	Ineffective	Inconclusive
4.22	Massage and aromatherapy	1	Nurses, setting NR	Can relieve pain through the stimulation of endorphins whilst using essential oils such as lavender can provide more pain relief through increased adrenocorticotropic hormone.	6 weeks 90 min	☒	☒	Aroma massage should be administered by professionals with specialized training. Particularly for hand massage, it can induce feelings of empathy towards the individual	Ø	Ø	Job-related stress
3.23	Gargle	1	Nurses; setting NR	15-20cm aromatic gargle solution daily.	10-15 seconds	☑	☒	Different essential oils can have different effect on an individual. For example, the use of ylang ylang has sedative effects on the nervous system.	Ø	Ø	Job-related stress
3.24	Bottle hung on chest	1	Nurses; setting NR	Lavender essential oil bottle hung on the right chest of participants.	4 days	☑	☒	There are differences in research views for inhalation therapy. Essentially, research argues that the main mechanism for aromatic therapy is between the olfactory and limbic system, where scent receptors in the nose send chemical-based messages to the brain's limbic region via the olfactory nerve.	Ø	Ø	Job-related stress

Intervention		Citations	Context	Mechanism					Outcomes		
			Macro-context	*Micro-context* (Resources)							
			Population, setting	**How/Why** the intervention works?	Duration	Content	Follow-up	Reasoning *(implicit/explicit presence of theory)*	Effective	Ineffective	Inconclusive
3.25	Inner forearm	1	Nurses; setting NR	5% solution of *Lavandula angustifolia* (3%) and *Salvia sclarea* (2%) in sweet almond oil as carrier. Five drops in the inner aspect of forearm was applied and rubbed with the opposite arm.	☒	☑	☒	When some essential oils are applied topically, it enables the skin to become more penetrable, which causes them to increase the effectiveness of other topical pharmaceuticals or via the use of a blended concoction.	Ø	Ø	Job-related stress
3.26	Storytelling (face-to-face, virtual)	1	Healthcare professionals, music and art therapist, staff administration, unlicensed healthcare workers.	Human oxytocin response can signal another person as safe, familiar, and trustworthy, and thus, this molecule initiates and motivates reciprocation.	☒	☒	☒	Stories can enable us to pay attention and allow use to become emotionally involved – moving us to make an action. This is particularly prominent for stories that have a dramatic arc	Ø	compassion fatigue, grief	Ø
3.27	Relaxation breathing; progressive muscle relaxation	1	Healthcare professionals: manufacturing, various, healthcare, secondary school, office- based	It encompasses a variety of strategies in aim to increase calmness and decrease stress. Since stress can contribute to physiological responses, relaxation can address these factors; for example, increased heart rate, muscle tension, shortness of breath, and subjective emotional experiences.	☒	☒	☒	Assists individuals to decrease anxiety and tension both physically and psychologically.	Ø	Ø	wellbeing

Intervention		Citations	Context	Mechanism					Outcomes		
			Macro-context	*Micro-context* (Resources)							
			Population, setting	**How/Why** the intervention works?	Duration	Content	Follow-up	Reasoning *(implicit/explicit presence of theory)*	Effective	Ineffective	Inconclusive
3.28	Neurofeedback-based relaxation	1	Healthcare professionals: manufacturing, various, healthcare, secondary school, office- based	Teaches aspects of self-control of the brain by providing feedback signals through brain waves. The electroencephalography records the neurofeedback treatment, where data is extracted and fed back to individuals using an online feedback tool as either audio, video or a combination of both.	☒	☒	☒	Enables individuals to interact, monitor, change, and manage their mental and physiological states.	Ø	Ø	wellbeing
3.29	Hatha Yoga	1	Nurses; ICU and critical care	Includes *pranayama* (breathing exercises), *asana* (physical movements), *shavasana* (deep relaxation).	6 sessions 15 min	☑	☒	It can improve one's breathing depth, chemoreceptive sensitivity, and reduces metabolic rate in healthy individuals. Moreover, the meditation aspect of yoga can facilitate hypo-metabolic states – *pranayama* does the opposite.	Ø	Ø	Stress

Intervention		Citations	Context	Mechanism					Outcomes		
			Macro-context	*Micro-context* (Resources)							
			Population, setting	**How/Why** the intervention works?	Duration	Content	Follow-up	Reasoning *(implicit/explicit presence of theory)*	Effective	Ineffective	Inconclusive
3.30	Physical exercise ((non-) workplace based, individually designed)	1	Healthcare professionals, nursing aides and assistant, no specification; setting NR	Led by an educated leader, included warming up, exercises of coordination, general strength exercises for back, arms, legs, and aerobic profiled activities with two or three intervals to increase hear rate of 120bpm, and stretching of major muscles. Music was played whilst activities were performed.	10 - 106 total hours	☒	☒	an extensive list of comorbidities can weaken an individual, which is often intensified in the state of exhaustion – leading to greater sense of burnout. Thus, physical exercises can be beneficial in terms of physiological changes and allow behavioural distraction from an stressful event (degrading the psychological impact of the event).	Ø	Ø	Occupational stress
3.31	Group Drug Counselling workshop and group consultation	1	Healthcare professionals, students, trainees; setting NR	Psychologically-focused group consultation and education workshop based on acceptance and commitment therapy, which included evidence-based treatment – Group Drug Counselling.	1 day – 8 weeks for 1.5 to 6 hours	☒	☒	Assists in breaking barriers of psychological barriers of the practitioner such as fears of judgements by others and discomfort in trying out new things.	Ø	Burnout, self-compassion, psychological wellbeing	Ø

	Intervention	Citations	Context<br>*Macro-context*<br>Population, setting	Mechanism<br>*Micro-context* (Resources)<br>**How/Why** the intervention works?	Duration	Content	Follow-up	Reasoning *(implicit/explicit presence of theory)*	Outcomes<br>Effective	Ineffective	Inconclusive
3.32	Narrative training	2	Physicians' music and art therapist, staff administration, unlicensed healthcare workers; medical/ICU/ critical care, paediatrics, psychiatry, radiology, surgery, gynaecology, multiple, unspecified, workplace, clinic, classroom, virtual meetings, NR	Wrote about emotional responses to patients and families, attachments to patients, attempt of viewing clinical situations from the patients and family member's perspectives. Narratives were then read along to one another, followed by a facilitated discussion.	☒	☒	☒	Illuminates' boundary conditions of expressive disclosure and discloses one's experiences to those who can benefit from it.	Ø	compassion fatigue, grief	Burnout, resilience
3.33	Knitting	1	Healthcare professionals, music and art therapist, staff administration, unlicensed healthcare workers.	Provided education on knitting instructions, materials in the basket, and different types of needles and yarns. Provided opportunity to knit during breaks or after a stressful event. Encouraged group knitting to enable time to debrief over the stressful event.	☒	☒	☒	Combination of repetitive tasks, cognitive/physical skills, and enjoyment to create a product. It also facilitates the combination of concentration, rhythm, and distance of oneself from the outside world, enabling the escape from negative issues.	Ø	compassion fatigue, grief	Ø

Intervention		Citations	Context	Mechanism					Outcomes		
			Macro-context	*Micro-context* (Resources)							
			Population, setting	**How/Why** the intervention works?	Duration	Content	Follow-up	Reasoning *(implicit/explicit presence of theory)*	Effective	Ineffective	Inconclusive
3.34	Music therapy	1	Healthcare professionals, music and art therapist, staff administration, unlicensed healthcare workers.	Music used open format – that is the improvisation of spontaneous musical and verbal expressions and creativity.	☒	☒	☒	Listening to music can initiates multiple aspects of the brain's cognitive processes. Music influences stress-related cognitive processes, which enables physiological responses.	Ø	compassion fatigue, grief	Ø
3.36	Mantram	1	Healthcare professionals	A silent, meaningful, self-selected, and repetitive word or phrase which aims to slow down behaviours and thoughts.	☒	☒	☒	Enables the ability of individuals to focus using one-pointed attention (focus on a single task at a time).	Ø	compassion fatigue, grief	Ø
3.35	Recreational Music-Making	1	Healthcare professionals, music and art therapist, staff administration, unlicensed healthcare workers; workplace, hospital clinic, classroom, virtual meetings,	Trained by a facilitator, utilised instruments including hand drums and an array of percussion instruments. Sessions included welcoming, introduction, overview, Yamaha Mind-Body Wellness exercise, ice-breaker activity, and utilisation of instruments.	☒	☒	☒	Accessible, enjoyable, and fulfilling group-based activity that enables individuals of all ages to unite regardless of their ethnicity, challenges, background, culture, abilities, or experiences. Used as a universal language to enhance interpersonal bonding by caring supportive guides that focuses on enabling individuals to achieve non-musical outcomes.	Ø	compassion fatigue, grief	Ø

	Intervention	Citations	Context	Mechanism					Outcomes		
			Macro-context	*Micro-context* (Resources)							
			Population, setting	**How/Why** the intervention works?	Duration	Content	Follow-up	Reasoning *(implicit/explicit presence of theory)*	Effective	Ineffective	Inconclusive
3.37	Art therapy	1	Healthcare professionals, music and art therapist, staff administration, unlicensed healthcare workers; workplace, hospital clinic, classroom, virtual meetings, NR	Can include art viewing, art making, healing quilts, clay mask making, and symbolic imagery.	☒	☒	☒	Can increase an individual's understanding of themselves and others, allows self-reflection, alter thinking patterns and behaviours, and reduce symptoms.	Ø	compassion fatigue, grief	Ø
3.38	Biofeedback-assisted relaxation (Only 1 study included workshop)	2	Healthcare professionals; setting NR	Utilises a 'brain wave synchronizer' to activate relaxation responses of the individual through the excitement of specific brain waves.	Twice weekly	☒	◙	Monitors physiological changes of emotions, thoughts and behaviours b providing information to individuals whilst they are practicing relaxation and cognitive changes – visualize effects of the biofeedback instrument.	Ø	Ø	Wellbeing, occupational stress, burnout
3.39	Intensive educational program in mindfulness, communicatio n, and self-awareness	2	Physicians, psychiatrists; surgery, medicine, primary and secondary care.	Silent retreat (at a retreat centre) where participants were engaged in a guided silent mindfulness practice for one day. Intensive training included didactic material, formal mindfulness meditation, and narrative and appreciative inquiry.	8 weeks to 10 months	☒	◙	Allows individuals to understand the inner and outward (social) components, personal resources, self-regulation, and prerequisites. Skills of communication can enable individuals to better regulate their emotions through reflection and maintain composure during tense situation.	Ø	Ø	resilience, burnout, occupation stress

Intervention		Citations	Context	Mechanism					Outcomes		
			Macro-context	*Micro-context* (Resources)							
			Population, setting	**How/Why** the intervention works?	Duration	Content	Follow-up	Reasoning *(implicit/explicit presence of theory)*	Effective	Ineffective	Inconclusive
4.40	Activities contributing comparisons, emotions, pushing away, thoughts, sensations (ACCEPTS)	1	Physicians; in medical/ICU/ critical care, paediatrics, psychiatry, radiology, surgery, gynaecology, multiple, unspecified	ACCEPTS is one of many tools used in dialectical behavioural therapy – develops the ability to use “wise-mind”, that is a metaphoric mental state between “logical” and “emotional” state	☒	☒	☒	Can develop emotion regulation as it incorporates mindfulness, focus on developing emotion-regulation skills, and emphasizes on the role of difficulties in ER. ER can hinder emotion-focused coping, distancing.	Ø	Ø	Burnout, resilience

Abbreviations: NR=not reported, min=Minutes, (-)=to, ☒=Not reported in citations, ☑=Reported in all citations, ◙=reported in some citations (inconsistencies), Ø= Nil, ICU=Intensive Care unit, ED=Emergency department, ER=Emotion Regulation.

Supplementary File 4 – Interview questions

Question No.	Question
1	Which interventions do you think would be most effective for individuals in a critical care context and why?
2	What strategies/interventions would you personally implement?
3	Why do you think these interventions would work for the critical care workforce in particular?
4	In your experience, what would you suggest are the barriers to implementing such interventions for people in the critical care workforce?
5	In your experience, are there any particular things which would enable them to more easily implement these interventions?
6	Are you aware of any interventions in relation to sleep, exercise, or triangulation that have been used and thought to be effective? If so, please describe. How would you apply these interventions?
7	In your opinion, do you think a combination of approaches would be more effective than one specific intervention? If so, can you suggest what combination of interventions might work for you and why?
8	Are there any interventions listed in the Ineffective table that you think should be considered/would be effective in a critical care context?
9	Are there any interventions you would have expected to be included as effective that were not listed in the CMOC tables?
10	In reading through the four tables, are the explanations of why interventions might work, logical?
11	In the tables provided, do the interventions listed provide adequate reasoning for their effectiveness? (*refer Reasonings Column)*
12	Anecdotally, organisational factors impact significantly on wellbeing and burnout. What would you suggest are some of the key organisational influences that impact significantly on individual's wellbeing and burnout?"
13	Is there anything else that you would like to add?
14*	In relation to those interventions that you believe would be effective, are there any theories/reasonings that you would change or elaborate on?
15*	What interventions/approaches would you suggest for managing a group that were resilient, but had high levels of emotional exhaustion?
16*	In your experience, what is the connection between depression and emotional exhaustion/burnout?

Abbreviations: No.=Number
*Questions 14 – 16 were added after the pilot interviews. See *Questioning Structure* subheading for further details.

Supplementary File 5 - Key FDC strategies adapted from Lincoln and Guba(Lincoln and Guba, 1986)

Rigour criteria	Purpose	Original strategies	Strategies applied in this review to achieve rigour
Credibility	Establish confidence that the reported results (from participant's perspective) are credible, true, and believable.	- Interviewing process and techniques<br>- Establishing investigator's authority.	- Used three pilot interviews.<br>- Ensured interviewers had required knowledge and skills.
Dependability	Ensure that the semi-structured interview findings are repeatable if it was conducted again in the same cohort of participants, coders, and context.	- Rich description of study methods<br>- Stepwise replication of the data	- Prepared an outline of the study including information sheet describing aims and endpoints<br>- Used an interviewer with immense experience in conducting semi-structured interviews.
Confirmability	Confidence that results obtained are corroborated or confirmed by other researchers.	- Reflexivity<br>- Triangulation	- Recorded (visual and audio) the interviews and had debriefing after interviews.<br>- Included triangulation techniques methodological, investigator and theoretical, and data source)
Transferability	Extend degree of results to be generalizable or transferrable to other settings and context.	- Purposeful sampling to form a nominated sample	- Used combination of purposive sampling techniques

Supplementary File 6 – Overview of expert responses summarised as Context-Intervention-Mechanism-Outcome Configuration (CMOC)

Contextual conditions	Intervention(s)/Factor(s)	Mechanisms	Outcome
	What interventions are effective for CCHPs		
Time efficient, logical, evidence-based, good reasoning to its effectiveness, participants would personally use, arguable that should be done out of work versus driven by the unit (engaging), easy for staff to attend. Considering using mindfulness on an individual level and use as daily practice (integrate into lifestyle)	Mindfulness and cognitive interventions	CCHPs require rationalization to fully understand and believe if an intervention is effective	Improve wellbeing, decrease burnout, develop self-awareness, emotional intelligence, and become disconnected from patient and work-related interactions upon leaving the workplace, increases resilience, able to make sound decision when calm (no improvision, thinking more clearly and emotionally in control).
Busy and stressful work environment.	Debriefing	talking to people with the same experiences or environment, discussing interventions, different perspective, having different personality types (i.e. outspoken and quiet)	Promotes job stress awareness, normalises the feelings of stress, encourages seeking assistance with workplace challenges, helps de-escalate issues in one's mind. De-escalates issues in one's mind, normalizes stressful experiences.
CCHPs are already resilient (hence their ability to work within such environments), logical, adequate reasoning. Need to provide safe environment (by trailed psychiatrists and supervised by psychologist) as they are vulnerable. CCHPs are already resilient (like challenges, stress, and adrenaline).	Resilience training	Building resilience can assist individuals in coping with heavy workload and recurrent stressful challenges	Logical nature (ability to rationalise), multifaceted, engaging in terms of their direct ability to implement learnt knowledge into clinical practice, mixed opinions on resilience training

Contextual conditions	Intervention(s)/Factor(s)	Mechanisms	Outcome
Logical, good reasoning, multifaceted approach, communication is part of the culture (i.e. communicating with stakeholders, patients, families)	Communication and stress management skills	Individuals who are not keen in mindfulness programs are likely to engage with this intervention.	Can directly implement within the workplace.
Practical, own positive experience, cohesive workforce	Emotional intelligence	Self-awareness	Contributes to self-awareness.
Practical, own positive experience, applicable and easy to do, most CCHP workforces can accommodate	Educational workshops	Individuals who are not keen in mindfulness programs are likely to engage with this intervention.	Can directly implement within the workplace.
Interventions that participants would personally implement,	All of the listed interventions above and include music, gratitude, observing others, speaking to patients and families, personal honesty and integrity	Multiforme approach	Able to relieve stress and burnout experiences
Previous experience where participants found effective for themselves.	Muscle relaxation, yoga	Alleviates physical tiredness	Able to manage stress
Opinions of effective interventions that are listed in the ineffective CMOC table			
Messuist went to the hospital and provided message to staff	Chair massage and music, Yoga, tai chi, mantram, aromatherapy, heart touch	Not identified	Enjoyable, felt valued by the organization
Needs to be done in a team, quick turnaround shifts	Art therapy interventions (i.e. jigsaw puzzles, craft, paintings, read, decorating, photography, vidual auditory, baking, house chores, singing)	Provides an outlet through creativity.	Helps to ‘turn off the mind’ after work.

Contextual conditions	Intervention(s)/Factor(s)	Mechanisms	Outcome
Similarities with mindfulness interventions	Neurolinguistic programming	Not identified	Difficult to determine efficacy due to only one study reporting on NLP
CCHPs may have a stressful experience – by talking to colleagues such as an informal debrief can help alleviate the stress	DBT – regular group meet sessions, telephone, support group, debriefing, coping groups	Reliving trauma within the support of such services	Stress reduction
CCHPs are provided with the opportunity to determine initial reactions, reflect, and improve oneself when encountered in 'real-life'.	Simulation training	Provides constructive feedback	Able to receive feedback and areas for improvements prior to the 'real' event.
Interventions not listed in the CMOC table			
Night shift staff, working overtime (unable to deny overtime work due to insufficient senior staffing such as accreditation to look after ventilated patient)	Understanding sleep patterns and psycadian patterns	Forcing oneself to sleep refutes the process of sleep	More of a consequence rather than an intervention to be considered
Need to have supportive management and colleagues – provide the time for professional supervision within work hours. Units should have entitlement for educational allowances.	Professional supervision	Not identified	Feel supported
Influences within the micro level			
Burnout is described a chronic condition, maintaining health and wellbeing should be within the workplace and extended outside the work environment, people have different ways of learning	"No one size fits all"	Depending on who introduces the intervention (may be effectively implemented if the person implementing is highly respectable).	Successful uptake

Contextual conditions	Intervention(s)/Factor(s)	Mechanisms	Outcome
suits individual's needs, effective interventions require sustainability, which comprises of the need to include the critical care workforce in the decision-making process of choosing an intervention to use. CCHP are becoming more isolated (fear of being a sign of weakness for caring their problems - perception).	combining interventions would be the preferred solution (i.e. mindfulness, cognitive restructuring; self-care, social support)	Enable the individuals to feel invested, leading to higher chances of successful implementation. If an intervention is imposed, individuals may feel less motivated. Consider looking at facets of personality	Addresses issues that the individual would like to improve on, efficient use of time, can be used at different circumstances
Instilling education (within the intervention) as a normal culture	Education	Increased knowledge	Education is useful, viable, and worthwhile for Continuous Professional Development
Some individuals prefer a one-to-one intervention with some direction on how to run the intervention	Individual interventions versus a group-based intervention	Group-based makes individuals feel that they are not alone, whereas one-to-one enables individuals feel that the intervention is personalised.	Both may be effective, personal preferences
Having different people running the intervention (especially if interventions bring out vulnerability).	Consistency	Individuals see the progress of their efforts	Individuals will continue with the intervention.
Influences within the macro level			
Culture of the organization and unit, burnout described as a chronic condition, maintaining wellbeing at work and within daily life.	Intervention is long-term	Needs someone to drive the program	CCHP feel guided in their journey

Contextual conditions	Intervention(s)/Factor(s)	Mechanisms	Outcome
Ongoing priorities within the organization, unit, and personal life, can consider technology use such as an application on the phone, podcast, or web-based interventions.	Time management/ efficiency	competing with demands that are difficult	Interventions would be useful for a short time period, but not long-term. staff leaving
Supportive managers in terms of time and supervision of patients during the intervention (if conducted within the unit), frequent exposures to death. Junior-heavy workforce	Support	Prioritizing staff members	Ease in implementation
CCHPs are strongly motivated, but lack resources, no staffing to provide the intervention, care-park problems for afternoon shift (unable to get to work before or on-time).	Lack of resources (interventions and financial constraints)	Not identified	Effective work ethic if they are able to combine resources with their motivation.
Management can be out of touch. Individual interventions can be effective, but still requires organizational factors to be addressed. Integration needs to be slow and achievable, prominent ‘lip-service’. Abundance of inertia (healthcare is looked at as a ‘soft-science’ (welfare). Having vicious cycle of bringing in agency staff but unable to afford permanent staff	System changes/ lifestyle changes	Can be overwhelming for some people, may look good on paper but may cause harm when applied.	Can lead to stress and burnout

Contextual conditions	Intervention(s)/Factor(s)	Mechanisms	Outcome
Co-designing (having champions) can be effective as it enables individuals to be invested in the intervention and likely to implement. When imposed, they are less motivated. Motivated and supportive colleagues amongst CCHPs, unable to determine the value and effects of the intervention	How the intervention is implemented	People want work-life balance – having interventions that they don't initiate can be a problem.	May not be invested in the intervention
Different settings such as rural versus metropolitan. In Australia, having positive work environment is paramount. If there is a large workforce within a unit, becomes difficult to accommodate to everyone's needs (especially if holding workshops). Reluctance to attend workshops after work hours. Unable to leave the unit due to factors such as deteriorating patients. Negative leadership qualities, lack of teamwork, isolation.	Work culture	Threat to the healthcare system even though there are excellent employees (CCHPs).	Inability to attend to interventions held within the workplace.
Societal expectations that is not achievable with the resources with have (pretentious – disingenuous), stigma	Recognition of staff wellbeing, health, and stress experiences	CCHPs want to feel valued, heard, and respected	Feeling valued
Lack of communication, management style, leaderships tyle, staffing, allocation	Support from management	Feeling unsupported	Discontinuation of the intervention

Abbreviation: CCHP=Critical Care Healthcare Professional, CMOC=context-mechanism-outcome configuration

Supplementary File 7 – Middle-range theories facilitating theory prepositions

Below presents a summary of the theory informing theory prepositions.

Theory proposition 1 – Skill acquisition theory

The skill acquisition theory denotes to how individuals progress in learning an array of skills, stemming from initial learning to broader aspects of advanced proficiency (VanPatten and Williams, 2014). The acquisition of skills can be observed within the everyday life. Individuals who wish to acquire a skill must learn the skill – either by trial-and-error, imitation, or seeking help from an instructor (Costa and Steffgen, 2015). Interventions that were chosen as effective by experts primarily concerned the process of skill acquisition. For example, learning stress management skills or even learning how to be mindful within mindfulness interventions. The individual's perception of their skill acquisition is paramount as it impacts on their strategic decisions (Costa and Steffgen, 2015). Individuals use their perception (of their skill acquisition) and make choices that concerns their strongest skills – and thus, how to take advantage of these (Costa and Steffgen, 2015). In other words, they have conscious awareness of their strongest skills (i.e. self-awareness) (Taie, 2014). As individuals transition from being attentive of implicitly learning the skill, they develop performance fluency (automatization), where the skill becomes fully spontaneous, effortless, errorless, fast, and often practiced without being aware of it anymore (Taie, 2014). It demonstrates the ability for individuals to acquire, practice, integrate, and reach a standpoint of automatically using skill into their daily life (i.e. clinical practice) and upon encountering challenging and stressful situations (Taie, 2014).

Theory proposition 2 – Self-determination theory

Having the full awareness on the credibility of an intervention is only one aspect of successful uptake of an intervention (Patrick and Williams, 2012). Rather intervention uptake is also influenced by the individual's motivation (autonomous decision) to change (Patrick and Williams, 2012). It engulfs the concepts and measures of autonomy, relatedness to others, perceived competence, and emphases on the social context to support optimal motivation (Patrick and Williams, 2012). Namely, autonomous regulation is a key factor, as the individuals tended to provide greater effort, persistence, engagement, and stability (Patrick and Williams, 2012). Thus, if autonomy can be facilitated, it is likely that interventions would be internalised (i.e. more valued over time) (Patrick and Williams, 2012). The self-determination theory also reflects that if interventions are implemented externally, for example, engaging in behaviours to receive rewards or avoid negative contingencies, it is likely that the intervention would not be internalized (Patrick and Williams, 2012).

Theory proposition 3 – Social capital theory

The social capital theory contends that resources, namely, social relationships cam develop and accumulate human capital (Machalek and Martin, 2015). It is further described as the feature of social relationship (i.e. trustworthy networks, social relations) that produces productive benefits (i.e. collaboration) (Luoma-aho, 2013; Machalek and Martin, 2015). The theory of social capital is closely assimilated with theories of social interactions and social networks (Luoma-aho, 2013). More specifically, bonding social capital focuses on network density, changing the identify to a 'we feeling', in-group cohesions, reciprocity, and norms. By facilitating an environment that is inclusive and accessible, it facilitates collaboration and access to resources, mitigating issues of power struggles (Luoma-aho, 2013).

Theory proposition 4 – Collaboration theory

The collaboration theory describes the collaborative interactions of individuals irrespective of their formal structures (i.e. manager-with-subordinates, or subordinate-with-subordinate)(Hurwitz and Adair, 2014). Collaboration in this theory is defined as on-going interpersonal interactions that disregards power imbalances, and with the purpose of achieving a common goal (Hurwitz and Adair, 2014). Heaton, Day, and Britten (2015) suggested that 'co-production' of knowledge enabled closer collaboration, facilitated an environment to seize opportunities and minimised barriers to reach successful translation of knowledge (Heaton et al., 2015). The process of co-production was purported to conceptualise relations and increase engagement between service providers and end users (Heaton et al., 2015).

Supplementary File 8 – Description of the revised program theory

Standpoint of social structures (Macro context)

Experts were asked about factors that facilitated and impeded the implementation of interventions. It enabled authors to determine the standpoint of social structures within critical care workforce, which permitted greater insight into mechanisms for consideration. The social structures reported surrounded organisational factors including work culture, high turnover rates (including large proportion of junior workforce), time management, financial constraints, and the lack of burnout recognition, resources, and support from management. Critical care workforces have busy and challenging work environments, which hinders time for employees to seek education for stress management. The lack of resources and financial support further impedes such opportunities, forcing the use of the individual's time. Thus, facilitating change requires specific allocated time and place for education so that individuals can learn and implement the intervention.

Experts also raised issues about conflicting priorities, where there exists an array of mandatory educational requirements to be completed, and this often means that additional interventions such as coping with burnout may not necessarily be at the top of an individual's priorities. All experts also agreed that work culture was a barrier to implementation. Cultural barriers included the interrelated aspects of negative team perception of the intervention, stereotypes (i.e. showing stress is a sign of weakness and incompetence), the lack of team motivation, acceptance of change, and the level of management support. Particularly, experts suggested that management support is a key indicator to successful implementation of the intervention. Experts suggested that management attempt to understand the views of employees to the implementation of a 'top-down' or a 'bottom-up' approach. Experts also noted that personal barriers can also be a significant contribution to hindering implementation. Examples given included feeling overwhelmed with additional tasks (intervention), emotional instability, personal views on the intervention, and the inability to find the right time at work. When focusing on emotional instability, experts suggest that expecting healthcare professionals to engage with the intervention can be challenging if they are already stressed, have low wellbeing, burnout, and are exhausted.

The components of '*Being aware of my surroundings'* and '*Don't ignore my problems at work'* were extracted from the umbrella review's program theory.

Components to facilitate successful outcome of interventions (Meso context)

Enablers proposed were a contrast of the barriers, which also included the facilitation of web-based programs and having change champions driving the program. These two components were highlighted *light green* in comparison to the *dark green* boxes. This is because the two components are identified as external enablers that facilitate successful uptake of the intervention. The darker green boxes comprising of (1) knowledge and skill development, (2) justifying interventions (rationalization), (3) accessibility and inclusivity, (4) collaboration, and (5) positive learning culture correlates to internal enablers that internally influences the intervention (micro level). It can be thought of as essential components required in the intervention for successful uptake by critical care healthcare components. Both *light* and *dark green* boxes are similar as they consider (contextual) influences from both the critical care healthcare professional and the environment. Further explanation of these components is described in the discussion section of the paper. The components of *'Tailoring to my needs'* and *'Ask me what I want'* were extracted from the umbrella review's program theory.

Context of effective interventions (Micro context)

Experts advised that mindfulness interventions and cognitive behaviour therapy facilitated components of self-awareness, emotional intelligence, and emotional detachment from work, which are all inter-related components that enhance wellbeing and decrease burnout. The contextual conditions of these interventions included flexibility, evidence-based, and the ability to be time efficient. Both interventions also provide strong logical reasoning on *how and why the intervention works*, which facilitates a logical process for critical care healthcare professionals to use and draw on conclusions from facts. Experts advised that debriefing can be effective as it promotes job stress awareness, de-escalates stressful events, normalises feelings of stress amongst employees, and encourages the pursue of support and assistance. The components of debriefing would be particularly useful for critical care as it directly addresses issues including reoccurring and intensified stressful situations, negative work culture, feelings of not being adequately supported, and individualised work conditions (i.e. working in silos). Experts had also suggested the use of resilience training and communication and stress management development as it provides skills such as adaptation to stressful situations and the ability to reinforce clear objectives and facilitate open communication. The context of these interventions is the ability to be engaging, logical, and multifaceted. Although experts voiced that resilience is a characteristic that is already present amongst critical care healthcare professionals, employing interventions to improve resilience can be beneficial due to the differences in resilience levels within the workforce. Improving emotional intelligence can be effective in protecting and reducing burnout experiences through a more contained reaction. The contextual conditions opinionated by experts were similar to those of mindfulness and cognitive behaviour therapy interventions. Experts indicated that they would personally or are currently using the above-mentioned interventions within their daily lives. The list extends to interventions such as self-compassion intervention, gratitude, relaxation techniques and more listed in the revised program theory (Figure 2) under the heading *Interventions to consider*.

Structured education was a vital component the encompassed all ‘effective’ interventions – evidence extracted from the umbrella review’s program theory.

References

Costa, A., and Steffgen, G. (2015). Contributors to Undergraduates' Perception of Skill Acquisition across Time. J. Educ. Train. Stud. 3:5, 26-34.

Every Nurse (n.d.). Critical Care Nurse. United States of America: EveryNurse, LLC. Available at: https://everynurse.org/careers/critical-care-nurse/ [Accessed October 12, 2020].

Heaton, J., Day, J., and Britten, N. (2015). Collaborative research and the co-production of knowledge for practice: an illustrative case study. Implement.Sci. 11:1, 1-10.

Hurwitz, M., and Adair, R. (2014). Collaboration Theory Stephanie Colbry Cabrini College. J. Leadersh. Educ. 13:14, 63-75.

Isa, K.Q., Ibrahim, M.A., Abdul-Manan, H.-H., Mohd-Salleh, Z.-A.H., Abdul-Mumin, K.H., and Rahman, H.A. (2019). Strategies used to cope with stress by emergency and critical care nurses. Br. J. Nurs. 28:1, 38-42.

Lincoln, Y.S., and Guba, E.G. (1986). But is it rigorous? Trustworthiness and authenticity in naturalistic evaluation. New. Dir. Eval. 1986:30, 73-84.

Luoma-aho, V. (2013). Corporate reputation and the theory of social capital. The handbook of communication and corporate reputation, 279-290.

Machalek, R., and Martin, M.W. (2015). Sociobiology and Sociology: A new synthesis. 892-898.

Patrick, H., and Williams, G.C. (2012). Self-determination theory: its application to health behavior and complementarity with motivational interviewing. Int. J. Behav. Nutr. Phys. Act. 9:1, 1-12.

Taie, M. (2014). Skill acquisition theory and its important concepts in SLA. Theory and practice in language studies 4:9, 1971-1976.

VanPatten, B., and Williams, J. (2014). Skill Acquisition Theory Robert DeKeyser, in *Theories in Second Language Acquisition*. (Routledge), 106-124.

World Health Organization (2008). Classifying health workers: Mapping Occpations to the International Standard Classification: International Standard Classificiation of Occupations. Geneva.
